# β‐Galactosidase‐Triggered Photodynamic Elimination of Senescent Cells with a Boron Dipyrromethene‐Based Photosensitizer

**DOI:** 10.1002/advs.202401012

**Published:** 2024-06-17

**Authors:** Jacky C. H. Chu, Blanca Escriche‐Navarro, Junlong Xiong, Alba García‐Fernández, Ramón Martínez‐Máñez, Dennis K. P. Ng

**Affiliations:** ^1^ Department of Chemistry The Chinese University of Hong Kong Shatin, N.T. Hong Kong China; ^2^ Instituto Interuniversitario de Investigación de Reconocimiento Molecular y Desarrollo Tecnológico Universitat Politècnica de València Universitat de València Valencia 46022 Spain; ^3^ Unidad Mixta de Investigación en Nanomedicina y Sensores Universitat Politècnica e València, Instituto de Investigación Sanitaria La Fe (IIS La Fe) Valencia 46026 Spain; ^4^ Unidad Mixta UPV‐CIPF de Investigación en Mecanismos de Enfermedades y Nanomedicina Universitat Politècnica de València, Centro de Investigación Príncipe Felipe Valencia 46012 Spain; ^5^ CIBER de Bioingeniería, Biomateriales y Nanomedicina (CIBER‐BBN) Instituto de Salud Carlos III Madrid 28029 Spain; ^6^ Department of Pharmacy The Affiliated Luohu Hospital of Shenzhen University Shenzhen University Shenzhen 518001 China

**Keywords:** boron dipyrromethene, β‐galactosidase, photodynamic therapy, senescent cells, singlet oxygen

## Abstract

Senescence is a cellular response having physiological and reparative functions to preserve tissue homeostasis and suppress tumor growth. However, the accumulation of senescent cells would cause deleterious effects that lead to age‐related dysfunctions and cancer progression. Hence, selective detection and elimination of senescent cells are crucial yet remain a challenge. A β‐galactosidase (β‐gal)‐activated boron dipyrromethene (BODIPY)‐based photosensitizer (compound **1**) is reported here that can selectively detect and eradicate senescent cells. It contains a galactose moiety connected to a pyridinium BODIPY via a self‐immolative nitrophenylene linker, of which the photoactivity is effectively quenched. Upon interactions with the senescence‐associated β‐gal, it undergoes enzymatic hydrolysis followed by self‐immolation, leading to the release of an activated BODIPY moiety by which the fluorescence emission and singlet oxygen generation are restored. The ability of **1** to detect and eliminate senescent cells is demonstrated in vitro and in vivo, using SK‐Mel‐103 tumor‐bearing mice treated with senescence‐inducing therapy. The results demonstrate that **1** can be selectively activated in senescent cells to trigger a robust senolytic effect upon irradiation. This study breaks new ground in the design and application of new senolytic agents based on photodynamic therapy.

## Graphical abstract

A tailor‐made photosensitizer based on boron dipyrromethene conjugated with a galactose moiety through a self‐immolative linker has been designed and synthesized. It exhibits a selective response toward the senescence‐associated β‐galactosidase, rendering it an efficient theranostic behavior for detection and photodynamic elimination of senescent cells in vitro and in vivo.

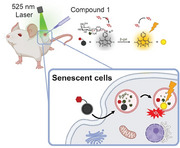

## 1 Introduction

Cellular senescence is a state in which cells stop proliferation and enter permanent cell growth arrest characterized by the expression of various cell cycle inhibitors, such as p16, p21, and p53.^[^ ^1^ ^]^ Besides, there are other common characteristic features of senescent cells, including a notable morphological change with irregular shape and increased size mainly attributed to the increased lysosomal compartments. As a result, senescent cells present a high lysosomal activity, particularly the overexpression of senescence‐associated β‐galactosidase (SA‐β‐gal). In addition, the senescent state is characterized by the activation of a dynamic and context‐dependent secretome known as the senescence‐associated secretory phenotype (SASP).^[^ ^2^, ^3^, ^4^ ^]^

Cellular senescence occurs naturally to deter the proliferation of stressed or damaged cells and trigger tissue repair to maintain the organism homeostasis.^[^ ^5^ ^]^ However, it has been reported that the accumulation of senescent cells disrupts normal tissue functions and promotes chronic inflammation and tumorigenesis, eventually leading to multiple age‐related dysfunctions and diseases.^[^ ^6^, ^7^ ^]^ Therefore, senescent cells have emerged as an attractive therapeutic target in age‐related disorders.^[^ ^8^ ^]^ From a different perspective, considering senescence as a tumor‐suppressing process, the guided induction of senescence can be applied for cancer treatment. However, despite the advantage of inhibition of tumor growth, patients still suffer from cancer relapse due to the harmful effect of senescence persistence.^[^ ^9^ ^]^ Hence, the use of senolytics that can specifically eliminate senescent cells has been proven to be an effective strategy in several pre‐clinical models.^[^ ^10^, ^11^ ^]^ Unfortunately, the associated side effects arised from the use of senolytics, such as navitoclax, fisetin, and the combined administration of dasatinib and quercetin have limited their translation to clinical practice.^[^ ^12^, ^13^, ^14^, ^15^ ^]^ Therefore, intensive efforts have been made in recent years to develop a new generation of senolytic drugs based on advanced prodrugs^[^ ^16^, ^17^ ^]^ and nanodevices^[^ ^18^, ^19^ ^]^ for targeting senescent cells while minimizing the negative outcomes.

In recent years, photodynamic therapy (PDT) has emerged as a promising alternative treatment modality against cancer, which has been successfully approved for the treatment of skin, bladder, lung, and breast cancers among others.^[^ ^20^, ^21^ ^]^ PDT induces tissue damage by reactive oxygen species (ROS) generated from the photochemical reactions of photosensitizers and molecular oxygen, killing cancer cells through apoptosis, necrosis, and/or other forms of regulated cell death.^[^ ^22^ ^]^ This is a multi‐step process that requires selective uptake of the photosensitizers by the target cells and tissues, followed by the local irradiation with light of a suitable wavelength to trigger the photochemical reactions that result in cell death and eventually tumor regression. Compared to the conventional anticancer therapies, PDT is relatively less invasive. It also has high spatiotemporal selectivity, which results in a highly site‐specific cytotoxic effect.^[^ ^23^ ^]^ To date, while approximately twenty photosensitizers have been approved or tested for clinical use for anticancer treatment, the low initial selectivity of photosensitizers is still a limitation of PDT.^[^ ^24^ ^]^ To address this issue, photosensitizers have been conjugated with a tumor‐targeting ligand and/or incorporated with an activatable component that is responsive toward tumor‐associated stimuli with a view to confining the photodynamic action to the target site.^[^ ^25^, ^26^, ^27^ ^]^

On this basis, we envisaged that enzyme‐activatable photosensitizers could be useful for eradication of senescent cells through controlled PDT. It is worth mentioning that the application of PDT for selective elimination of senescent cells remains little studied. While a number of β‐galactosidase (β‐gal)‐activatable photosensitizers have been reported, most of them are tested in β‐gal‐overexpressing cells or in engineered models based on *lac*Z gene transfection, which are not directly related to a real scenario of senescence.^[^ ^28^, ^29^, ^30^, ^31^, ^32^ ^]^ To the best of our knowledge, only a handful of photosensitizers that can be activated by SA‐β‐gal in senescent cells have been reported so far, including a methylene blue‐based molecular agent,^[^ ^33^, ^34^ ^]^ the selenium‐substituted dicyanomethylene‐4H‐pyran KSL0608‐Se,^[^ ^35^ ^]^ a black hole quencher‐conjugated boron dipyrromethene (BODIPY),^[^ ^36^ ^]^ and a nanophotosensitizer formed by self‐assembly of a self‐quenched zinc(II) phthalocyanine dimer,^[^ ^37^ ^]^ all containing a β‐galactose moiety as a β‐gal substrate. Among these photosensitizers, only KSL0608‐Se was evaluated for its in vivo properties. In naturally aged mice, this photosensitive senolytic prodrug could prevent the upregulation of age‐related senescent cell markers and SASP factors. For the other photosensitizers, their senolytic effect by PDT has not been studied at the animal level.

We report herein another BODIPY‐based photosensitizer that can be selectively activated by SA‐β‐gal to simultaneously detect and eradicate senescent cells. Compared with our previously reported analog,^[^ ^36^ ^]^ which requires the conjugation with a black hole quencher via a self‐immolative linker through a lengthy synthetic route, the molecular design of this compound is much simpler, yet the therapeutic efficacy is remarkably higher. To move one step forward toward clinical translation, we also examined the in vivo PDT effect of this photosensitizer in a clinically relevant scenario, using a palbociclib‐induced senescent melanoma model for evaluation of its senolytic effect. Our findings represent a significant advancement in the field of senolytic therapy, providing insights for the design and application of innovative senolytic agents based on PDT.

## 2 Results and Discussion

### 2.1 Molecular Design, Synthesis, and Characterization

**Figure 1:**
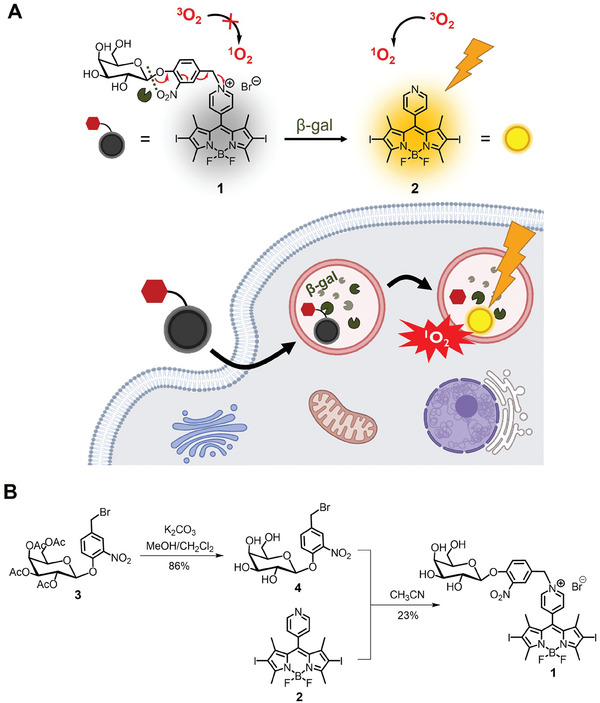
A) Schematic illustration of the photodynamic eradication of senescent cells by the SA‐β‐gal‐activatable BODIPY‐based photosensitizer **1**. B) Synthetic scheme of **1**.

This SA‐β‐gal‐activatable photosensitizer is comprised of three components, namely a pyridinium BODIPY‐based photosensitizer, a self‐immolative nitrophenylene linker, and a galactose moiety as a β‐gal substrate. A BODIPY was chosen as the photosensitizing unit due to its well‐established chemistry and tunable spectroscopic and photophysical properties.^[^ ^38^ ^]^ Previous studies have shown that the fluorescence emission of meso‐4‐pyridinyl BODIPYs is quenched after *N*‐alkylation at the pyridine moiety through a photoinduced electron transfer (PET) process.^[^ ^39^, ^40^ ^]^ This quenching effect is also effective for the iodinated analogs.^[^ ^41^ ^]^ Similarly, a ruthenium(II) polypyridyl moiety has also been used to cage a meso‐4‐pyridinyl BODIPY and its diiodo analog.^[^ ^42^ ^]^ Upon green light irradiation, free meso‐4‐pyridinyl BODIPYs are released to restore their fluorescence emission and singlet oxygen formation (for the diiodo derivative). Inspired by these results, we conjugated a galactose moiety to a meso‐4‐pyridinyl BODIPY through a self‐immolative nitrophenylene linker by *N*‐alkylation. It was expected that the photoactivities of this photosensitizer (compound **1**) would be quenched through a PET mechanism due to the presence of the pyridinium moiety. Upon interactions with the SA‐β‐gal in senescent cells, the β‐galactosidic bond would be cleaved, followed by rapid self‐immolation through 1,6‐elimination to generate the highly fluorescent and photocytotoxic photosensitizer **2**, thereby achieving selective eradication of the senescent cells (**Figure** **1**A).

Figure 1B shows the synthetic scheme of this novel SA‐β‐gal‐activatable photosensitizer. The galactosylated benzyl bromide **3** was prepared according to our previously reported procedure.^[^ ^27^ ^]^ It was then hydrolyzed with K_2_CO_3_ in a mixture of MeOH and CH_2_Cl_2_ (1:4 v/v) to remove the acetyl groups to give compound **4**, which was then treated with the diiodo pyridinyl BODIPY **2**.^[^ ^41^ ^]^ to afford the target conjugate **1**. Both **1** and **4** were characterized with various spectroscopic methods (Figures S1–S4, Supporting Information, which also includes the experimental details).

### 2.2 Photophysical Properties and Enzymatic Activation

**Figure 2:**
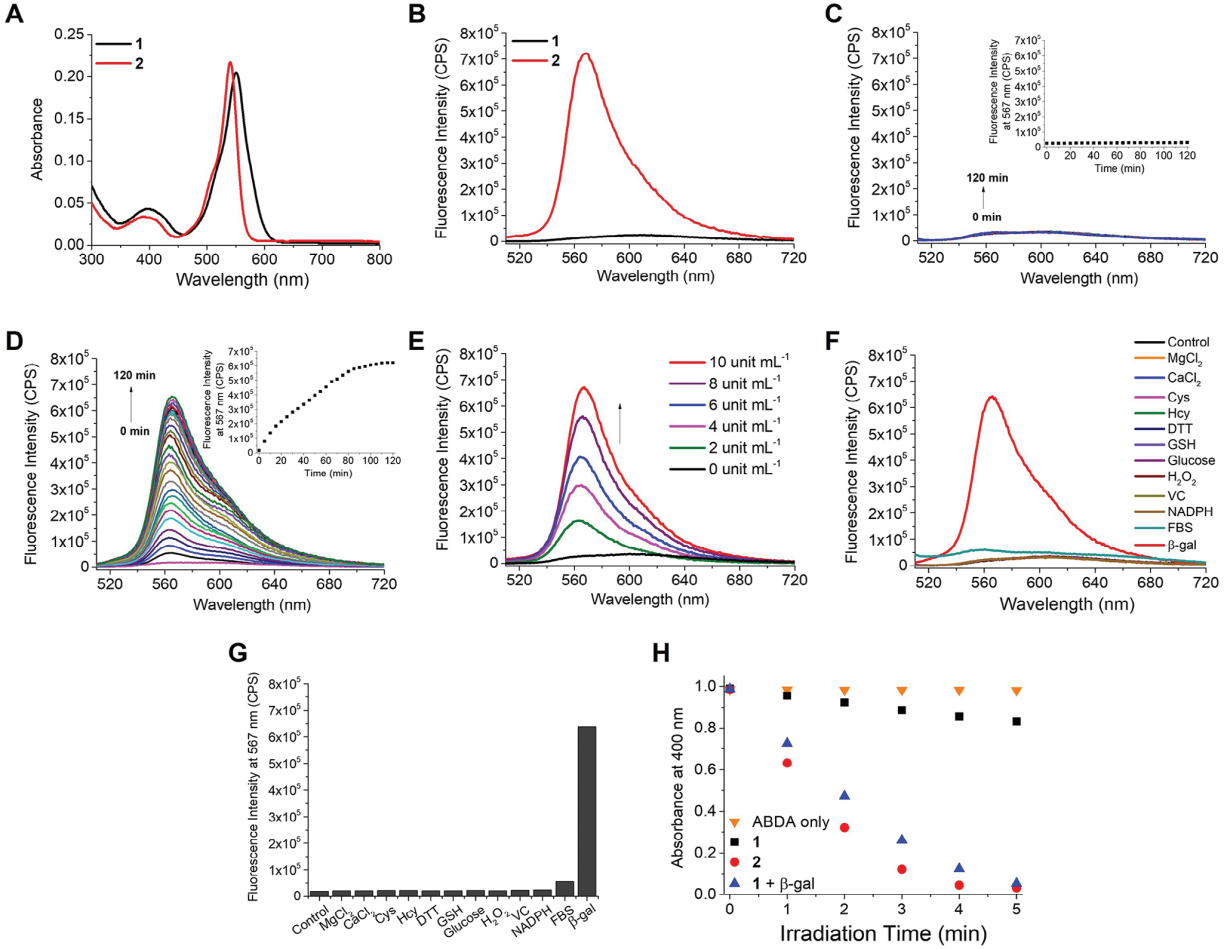
A) Electronic absorption and B) fluorescence spectra of **1** and **2** (2 µm). Change in fluorescence spectrum of **1** (2 µm) in the C) absence and D) presence of β‐gal (10 unit mL^−^^1^) over 2 h. The inset shows the change in fluorescence intensity at 567 nm over 2 h. E) Fluorescence spectra of **1** (2 µM) after the treatment with various concentrations of β‐gal (0‐10 unit mL^−^^1^) for 2 h. F) Fluorescence spectra and G) the corresponding fluorescence intensity at 567 nm of **1** (2 µm) after the treatment with various species (1 mm), FBS (10% v/v in RPMI 1640 medium), or β‐gal (10 unit mL^−^^1^) for 2 h. H) Comparison of the rates of decay of ABDA (initial concentration = 60 µm) sensitized by **1** (2 µm), with or without pre‐treatment with β‐gal (10 unit mL^−^^1^) for 2 h, as well as **2** (2 µm) upon light irradiation (λ > 515 nm). The solvent was PBS at pH 7.4 with Tween 80 (0.1% v/v) for all these measurements unless stated otherwise. All the fluorescence spectra were recorded upon excitation at 500 nm.

The electronic absorption and fluorescence spectra of **1** were recorded in phosphate‐buffered saline (PBS) at pH 7.4 with Tween 80 (0.1% v/v) added to improve its water solubility. The results were compared with those of **2**. As shown in **Figure** **2**A, an intense π→π* absorption was observed for **1** at 550 nm, which was slightly red‐shifted compared with that of **2** (at 540 nm). Upon excitation at 500 nm, **1** showed a weak fluorescence band at 600 nm with a fluorescence quantum yield (Φ_F_) of 0.038 relative to fluorescein in NaOH(aq) (0.1 m, pH 13, Φ_F_ = 0.925),^[^ ^43^ ^]^ which could be attributed to the quenching effect of the pyridinium moiety through PET. In contrast, **2** emitted strongly at 567 nm with a Φ_F_ value of 0.41 (Figure 2B). The remarkable difference shows that the fluorescence emission of the meso‐4‐pyridinyl BODIPY core can be successfully caged by the *N*‐alkylated substituent. All these spectroscopic and photophysical data are summarized in **Table** **1**.

**Table 1:** Electronic absorption and photophysical properties of **1** and **2** in PBS at pH 7.4 with Tween 80 (0.1% v/v).

Compound	λ_abs_ (nm) (log ε)	λ_em_ (nm)^a)^	Φ_F_ ^b)^	Φ_Δ_ ^c)^
**1**	398 (4.21), 550 (5.20)	600	0.038	0.12
**2**	392 (4.19), 540 (5.22)	567	0.41	0.60

^a)^ Excited at 500 nm;

^b)^ Relative to fluorescein in NaOH(aq) (0.1 m, pH 13, Φ_F_ = 0.925);

^c)^ Relative to rose bengal (Φ_Δ_ = 0.75).

We then studied the activation of **1** by β‐gal by monitoring the change in its fluorescence spectrum both in the absence and presence of β‐gal (10 unit mL^−1^) over a period of 2 h. The results showed that while the fluorescence intensity remained weak and unchanged in the absence of β‐gal (Figure 2C), the fluorescence band at 567 nm was dramatically intensified along with time upon addition of β‐gal, and the intensity reached the maximum after ≈2 h (Figure 2D). Under these conditions, the pseudo first‐order rate constant was determined to be 0.020 min^−1^ (Figure S5A, Supporting Information). To further investigate the enzymatic kinetics, the fluorescence intensities of the mixtures of different concentrations of **1** and β‐gal (10 unit mL^−1^) were recorded at different time points, and then the data were fitted into the Lineweaver‐Burk plot (Figure S5B, Supporting Information). According to the Michaelis‐Menten equation, the Michaelis constant *K_M_* was determined to be 1.42 µm, which is lower than those of the previously reported β‐gal‐activatable methylene blue‐based photosensitizer (4.32 µm),^[^ ^33^ ^]^ and the commercial fluorescein di‐β‐D‐galactopyranoside (10.2 µm),^[^ ^44^ ^]^ showing that **1** has a relatively high affinity toward β‐gal. As expected, the fluorescence intensity was increased with the concentration of β‐gal used (from 0 to 10 unit mL^−1^) (Figure 2E). All these results showed that the fluorescence emission of **1** could be activated by β‐gal.

To examine whether the activation would be affected by common interfering species in the biological systems, we monitored the change in the fluorescence spectrum of **1** in the presence of MgCl_2_, CaCl_2_, cysteine (Cys), homocysteine (Hcy), dithiothreitol (DTT), glutathione (GSH), glucose, H_2_O_2_, vitamin C (VC), or nicotinamide adenine dinucleotide phosphate (NADPH) (all at 1 mm) in PBS at pH 7.4 with Tween 80 (0.1% v/v), as well as fetal bovine serum (FBS) (10% v/v) in Roswell Park Memorial Institute (RPMI) 1640 medium. These species are generally present in the cellular environment and cell culture media and have been used for studying the selectivity of β‐gal.^[^ ^33^, ^36^ ^]^ The use of FBS, which is a complex mixture of growth factors, lipids, proteins, trace elements, vitamins, hormones, etc. enables the study of the effect of complex biomolecules. The results showed that none of them could induce a significant change in the spectrum, which was in large contrast with the remarkable activation effect of β‐gal (Figure 2F,G). The non‐responsive property of **1** toward the aforementioned serum‐containing culture medium was also demonstrated using high‐performance liquid chromatography (HPLC). In the absence of β‐gal, the chromatogram of **1** remained unchanged, displaying a single sharp peak, over a period of 4 h at 37 °C (Figure S6, Supporting Information), showing that it is stable under these conditions. Upon treatment with β‐gal (10 unit mL^−1^) for 2 h in this culture medium, the fluorescence emission of **1** could also be activated (Figure S7, Supporting Information) as in PBS (Figure 2E).

To confirm that the activation of **1** by β‐gal led to the formation of **2**, the mixture of **1** (2 µM) and β‐gal (10 unit mL^−1^) in PBS with Tween 80 (0.1% v/v) was analyzed by liquid chromatography – mass spectrometry (LCMS). As shown in Figure S8 (Supporting Information), a new signal with a retention time of 39.5 min appeared in the chromatogram after 1 h, which could be attributed to **2** by comparing their retention times and examining its electrospray ionization (ESI) mass spectrum (*m*/*z* = 577 for the [M+H]^+^ ion), while the signal of **1** at 23.2 min was also observed with a lower intensity. The former became the exclusive signal in the chromatogram after 2 h, confirming that the conversion was completed within this period of time.

Apart from monitoring the change in fluorescence emission, the effect of β‐gal on the singlet oxygen generation efficiency of **1** was also investigated using 9,10‐anthracenediyl‐bis(methylene)dimalonic acid (ABDA) as a singlet oxygen scavenger.^[^ ^45^ ^]^ As shown in Figure 2H, the absorbance of the ABDA's absorption at 400 nm remained unchanged in the absence of any photosensitizer. Upon light irradiation (*λ* > 515 nm), **1** could only slightly reduce the absorbance, showing that only a small amount of singlet oxygen was generated. The singlet oxygen quantum yield (Φ_Δ_) was determined to be 0.12 relative to rose bengal (Φ_Δ_ = 0.75).^[^ ^46^ ^]^ However, the rate of decay of ABDA was substantially faster when **1** was pre‐treated with β‐gal (10 unit mL^−1^) for 2 h. Under this condition, the rate was comparable with that by using **2** as photosensitizer, for which the value of Φ_Δ_ was determined to be 0.60 (Table 1). These results clearly indicated that the singlet oxygen generation of **1** could also be caged by the *N*‐alkylated substituent and be almost fully restored upon the action of β‐gal.

### 2.3 In Vitro Photocytotoxicity

**Figure 3:**
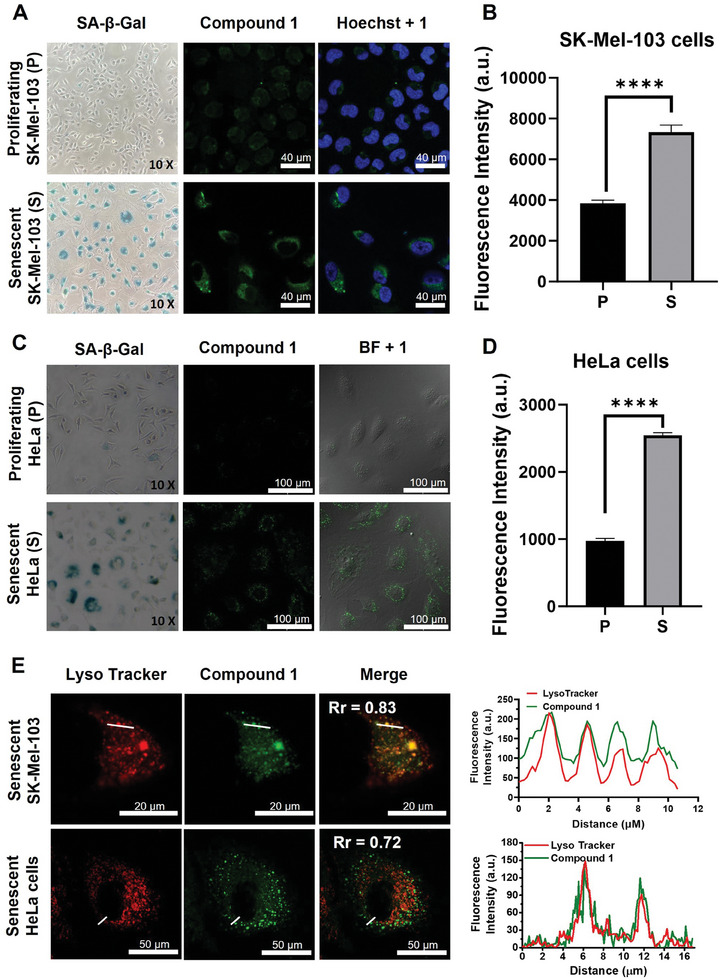
Confocal images of proliferating and senescent A) SK‐Mel‐103 and C) HeLa cells. From left to right: X‐gal stained images, fluorescence images of the cells after incubation with **1** (2 µm) for 2 h, and the merged images (with the Hoechst‐stained or bright‐field images). Scale bar represents 40 µm for SK‐Mel‐103 cells and 100 µm for HeLa cells. B,D) Quantification of the corresponding fluorescence intensities. Data are reported as the mean ± standard error of the mean (SEM) of three independent experiments, and statistical significance was assessed by t‐test (^****^ *p* <0.0001). E) Confocal fluorescence images of senescent SK‐Mel‐103 and HeLa cells after costaining with **1** (in green) and LysoTracker (in red). Areas of co‐localization appear in yellow/orange in the Merge panel. Pearson's correlation coefficient (Rr) reported therein represents a correlation between the pixel intensity of **1** and that of the tracker in the enlarged image. The graphs on the right represent fluorescence intensity profiles along the white line drawn through the cells. Scale bar represents 20 µm for SK‐Mel‐103 cells and 50 µm for HeLa cells.

Being encouraged by these results, we further examined the β‐gal‐triggered response of **1** in vitro using two different drug‐induced senescent models, namely human melanoma SK‐Mel‐103 cells treated with the CDK4/6 inhibitor palbociclib (5 µm, 7 days) and human cervical adenocarcinoma HeLa cells treated with the anticancer drug doxorubicin (50 nm, 3 days).^[^ ^36^, ^47^ ^]^ After the respective treatment, both cell lines became senescent and exhibited an obvious morphological enlargement and an increased SA‐β‐Gal activity confirmed by the X‐gal staining assay (**Figure** **3**A,C, the leftmost column).^[^ ^48^ ^]^ The increased level of β‐gal in the senescent HeLa cells was also verified with the commercial fluorogenic probe C_12_FDG (Figure S9A, Supporting Information).^[^ ^48^ ^]^

After the establishment of the senescent cell models, the cells were incubated with **1** (2 µm) for 2 h and then examined by confocal microscopy and flow cytometry. It was found that the intracellular fluorescence for the senescent SK‐Mel‐103 and HeLa cells was much stronger than that for the proliferating counterparts (Figure 3A,C). The intensity was determined to be 1.9 and 2.6‐fold stronger, respectively, by flow cytometry (Figure 3B,D). Further post‐incubation of the senescent HeLa cells in a fresh medium for 2 and 4 h did not result in further increase in fluorescence intensity (Figure S9B, Supporting Information), which may be attributed to the lysosomal degradation and/or exocytosis of these small molecular photosensitizers.^[^ ^49^ ^]^ All these results showed that **1** could be activated by the endogenous SA‐β‐gal in senescent cells.

The subcellular localization of **1** in both senescent cell lines was further investigated. As shown in Figure 3E, the fluorescence signal due to the activated species (i.e., BODIPY **2**) overlapped well with that of the LysoTracker. The Pearson´s correlation coefficients (Rr) were determined to be 0.8 ± 0.1 and 0.7 ± 0.1 for senescent SK‐Mel‐103 and HeLa cells, respectively. However, the signal did not overlap well with that of MitoTracker and ER‐Tracker (Figure S10, Supporting Information). Hence, the activation showed a high degree of lysosomal localization, which could be explained by the fact that β‐gal activity is found in the lysosomes.

**Figure 4:**
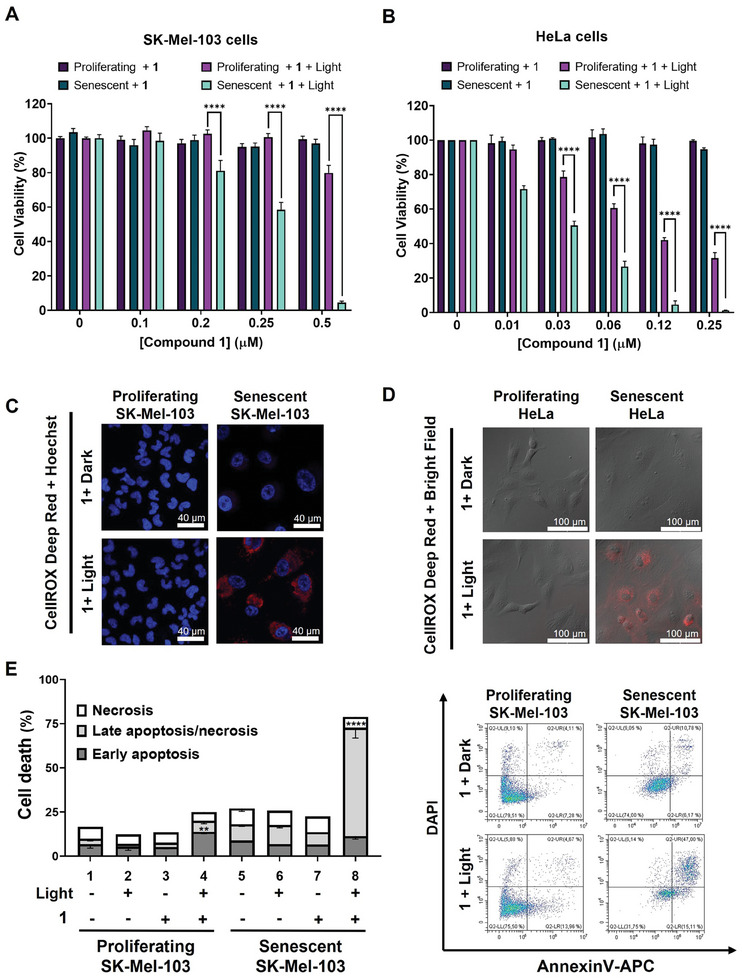
Cytotoxicity of **1** against proliferating and senescent A) SK‐Mel‐103 and B) HeLa cells in the absence and presence of light irradiation [for SK‐Mel‐103 cells: *λ* > 475 nm, 14.3 mW cm^−2^, 25.7 J cm^−2^ (for 30 min); for HeLa cells: *λ* > 515 nm, 25.5 mW cm^−2^, 30.6 J cm^−2^ (for 20 min)]. Data are reported as the mean ± SEM of three independent experiments, and statistical significance was assessed by two‐way ANOVA followed by Dunnett's post‐test (^****^ *p* <0.0001). Intracellular ROS production as reflected by the fluorescence of the oxidized form of CellROX Deep Red Reagent in proliferating and senescent C) SK‐Mel‐103 and D) HeLa cells after being treated with **1** (0.5 µm for SK‐Mel‐103 cells and 2 µm for HeLa cells) for 2 h, followed by the dark or light treatment with the aforementioned source for 5 min. Scale bar represents 40 µm for SK‐Mel‐103 cells and 100 µm for HeLa cells. E) Cell apoptosis/necrosis assay by flow cytometry for SK‐Mel‐103 cells after the treatment with **1** both in the absence and presence of light irradiation. (Left) Quantification of early apoptotic, late apoptotic/necrotic, and necrotic cells. Percentages are expressed as the mean ± SEM of at least three independent experiments, and statistical significance was assessed by two‐way ANOVA and Dunnett´s post‐test (^**^ *p* <0.01, ^****^ *p* <0.0001). (Right) Representative dot plot diagrams for apoptotic cell population with Annexin V‐APC and DAPI staining.

The in vitro photodynamic activity of **1** was then evaluated against the proliferating and senescent SK‐Mel‐103 and HeLa cells. The cells were incubated with various concentrations of **1** for 2 h, followed by dark or light treatment. The cell viabilities under the different conditions were then determined. In the absence of light irradiation, cell viability was virtually unchanged for all the cell lines, indicating that **1** showed negligible dark cytotoxicity (**Figure** **4**A,B). In contrast, the cell viability dropped significantly upon light irradiation, particularly for the senescent cells, for which the half‐maximal inhibitory concentrations (IC_50_ values) were determined to be 0.27 and 0.03 µm for senescent SK‐Mel‐103 and HeLa cells, respectively. These values are much lower than the IC_50_ values of the previously reported β‐gal‐activated methylene blue (1 µm).^[^ ^33^, ^34^ ^]^ and BODIPY (>10 µm)^[^ ^36^ ^]^ based photosensitizers. For the proliferating cells, while the cell viability was virtually unchanged for SK‐Mel‐103 cells when up to 0.25 µm of **1** was used, the value was dropped to ca. 30% for HeLa cells at this concentration. The significantly different photocytotoxicity could be attributed to the different cell type that could exhibit different inherent β‐gal activity as we found previously.^[^ ^36^ ^]^ In fact, the blue stain of X‐gal was more visible for the proliferating HeLa cells compared with the proliferating SK‐Mel‐103 cells as shown in Figure 3A,C (the upper leftmost image). Hence, the significant photocytotoxicity of **1** against the proliferating HeLa cells could be due to their intrinsic β‐gal level. In addition, the study of the photocytotoxicity of **1** against the two cell lines was carried out independently using two different light sources. While these results for the two cell lines could not be directly compared, the trend was consistent for both cell lines, which could demonstrate the reliability and validity of the findings.

To further study the photodynamic activity of **1**, the ROS generation efficiency in senescent cells was also investigated. After incubation with **1** (0.5 µm for SK‐Mel‐103 cells and 2 µm for HeLa cells) for 2 h, the cells were treated with the CellROX Deep Red Reagent as a ROS probe, which emits a bright red fluorescence signal upon oxidation by ROS.^[^ ^50^ ^]^ As shown in Figure 4C,D, the fluorescence was negligible in both proliferating and senescent cells in dark. However, upon light irradiation for 5 min, a relatively strong red fluorescence was observed in the senescent SK‐Mel‐103 and HeLa cells, but not in the proliferating cells under these experimental conditions. The corresponding quantified intensities are given in Figure S11 (Supporting Information) for quantitative comparison.

Moreover, we also studied the cell death mechanism of **1** against SK‐Mel‐103 cells using an annexin V‐APC and DAPI costaining method. The flow cytometric data are summarized in Figure 4E. While most of the cells remained viable for both proliferating and senescent cells in dark, light treatment (*λ* > 475 nm, 14.3 mW cm^−2^, 25.7 J cm^−2^) for 30 min induced 11% early apoptosis (Annexin+/DAPI‐), 61% late apoptosis/necrosis (Annexin+/DAPI+), and 6% necrosis (Annexin‐/DAPI+) for the senescent cells, which confirmed that apoptosis is the main cell death mechanism. For the proliferating cells, the cytotoxicity effect of **1** was not significant. These results further confirm that **1** can be activated in senescent cells inducing cell death after light irradiation.

### 2.4 In Vivo PDT Efficacy

Given the ability of **1** to selectively eliminate senescent cells in vitro, we moved one step forward to evaluate its in vivo photodynamic activity against a clinically relevant palbociclib‐induced senescent melanoma mouse model. Melanoma represents a significant clinical challenge due to its aggressiveness and multidrug resistance. PDT has emerged as a potential treatment modality for this cancer with ongoing clinical trials to evaluate its efficacy (e.g., NCT00862901, NCT03110159, and NCT00007969). Additionally, senescence induction is being explored as a tumor suppressor in clinical trials using melanoma as a model (e.g., NCT02202200 and NCT04720768).^[^ ^51^ ^]^ However, the accumulation of senescent cells may promote angiogenesis, chronic inflammation, and cancer recurrence. To overcome these negative effects, strategies involving senescence‐inducing chemotherapy followed by senolytic therapy are being developed. However, traditional senolytics face limitations due to the associated toxicities, leading to a strong demand of effective approaches for selective elimination of senescent cells, which was one of the reasons triggering this study.

**Figure 5:**
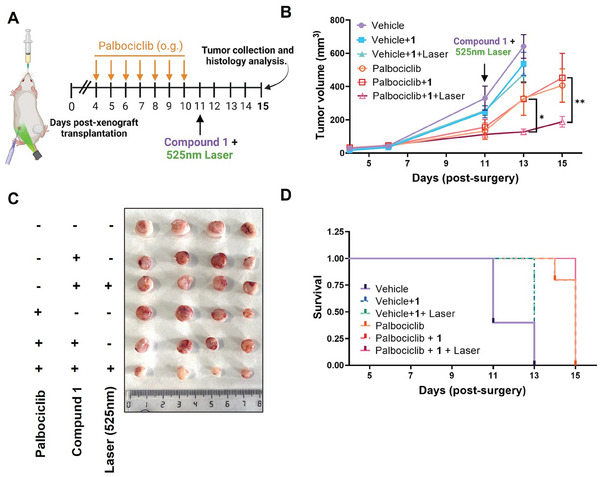
A) Athymic nude female mice were subcutaneously injected with SK‐Mel‐103 melanoma cancer cells in both flanks (n = 7 tumors per group). When the tumor volume reached ca. 50 mm^3^, either vehicle or palbociclib treatment started over a period of 7 days. Once tumor growth was arrested, a single dose of **1** was intratumorally administered and after 2 h, the tumor was irradiated (λ = 525 nm, 500 mW cm^−2^, 150 J cm^−2^) for 5 min. B) Change in tumor volume of SK‐Mel‐103 xenografts after the different treatments. Data are reported as the mean ± SEM. Statistical significance was assessed by two‐way ANOVA followed by Tukey's post‐test (^**^ *p* < 0.01, ^***^ *p* < 0.001). C) Photograph of representative tumor samples for each treatment. Scale bar = 1 cm. D) Kaplan‐Meier curve during the experimental period in response to the treatments. All the treatments enhanced the survival of the mice.

The timeline of the in vivo study is shown in **Figure** **5**A. All mice were treated in strict accordance with the Ethical Committee for Research and Animal Welfare Generalitat Valenciana, Conselleria dÀgricultura, Medi ambient Canvi climàtic i Desenvolupament Rural (2022 VSC PEA 193). SK‐Mel‐103 cells were first subcutaneously injected in athymic nude mice. When the tumor volume reached ca. 50 mm^3^ on day 4, daily therapy of palbociclib (50 mg kg^−1^ via oral gavage) was initiated to induce senescence in the tumor. For comparison, the mice were fed only with the vehicle (50 mm sodium lactate at pH 4.5). After the treatment with palbociclib for 7 days, significant reduction in tumor size was observed compared with the non‐palbociclib‐treated control group, and the induction of senescence at this point was confirmed by the increased X‐gal staining in the whole tumor (Figure S12A, Supporting Information). Once tumor growth was arrested, the palbociclib treatment was ended and the animals were divided into different groups. Animals with a proliferating tumor (i.e., not being treated with palbociclib) were referred as group (1) vehicle, (2) vehicle + **1**, and (3) vehicle + **1** + laser. Animals with a senescent tumor (i.e., being treated with palbociclib) were referred as group (4) palbocilib, (5) palbociclib + **1**, and (6) palbociclib + **1** + laser. On day 11, **1** was intratumorally injected [40 nmol in 200 µL of deionized water containing 2.5% (v/v) dimethylsulfoxide (DMSO)]. After 2 h, the tumors (for group 3 and 6) were irradiated for 5 min (λ = 525 nm, 500 mW cm^−2^, 150 J cm^−2^) and the tumor growth was monitored over the next few days. Non‐irradiated tumors treated with **1** (i.e., group 2 and 5) were also included as a control. Remarkably, treatment with **1** in the presence of light irradiation resulted in significant tumor regression for the palbociclib‐induced senescent tumor (Figure 5B,C). In contrast, for the vehicle groups with a proliferating tumor, treatment with **1** did not exhibit any sign of tumor size reduction regardless of whether light irradiation was applied, corroborating the need of SA‐β‐gal for activating the PDT effect of **1**. For the palbociclib and palbociclib + **1** control groups, the tumor inhibition effects were comparable and weaker than that of the complete treatment (i.e., group 6), showing that light irradiation is also essential to trigger the therapeutic effect of **1**. It is worth mentioning that for all the three vehicle groups, some of the mice needed to be sacrificed between day 11 and 13 due to the exponential growth of the tumor. Similarly, some animals in palbociclib and palbociclib + **1** groups also needed to be sacrificed on day 14 or 15 due to the proliferation of tumor cells after ceasing the treatment with palbociclib. In contrast, the animals receiving the complete treatment could be maintained alive to the end point of the experiment (Figure 5D). Moreover, the mice did not have significant body weight loss during the various treatments, suggesting that the treatments did not induce notable toxicity (Figure S12B, Supporting Information).

**Figure 6:**
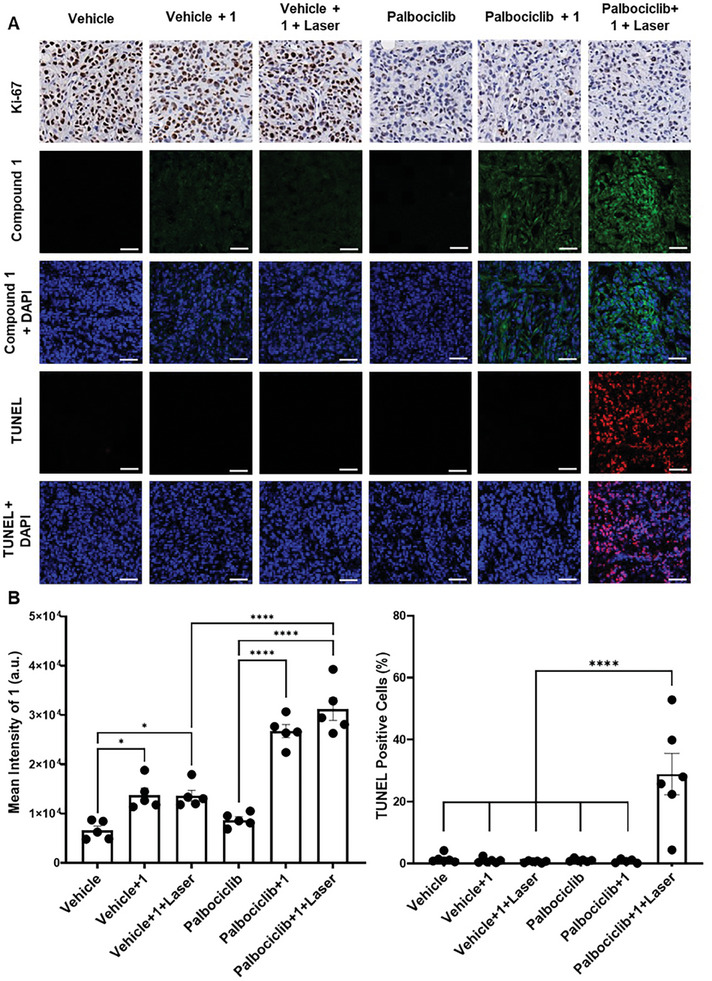
A) Representative histological images of SK‐Mel‐103 tumors at the end of different treatments stained for Ki67, BODIPY‐associated fluorescence from **1**, and TUNEL staining. Scale bar = 50 µm. B) Percentage of (left) **1**‐stained and (right) TUNEL‐positive cells in tumors (*n* ≥ 5 tumors per group). Data represent the mean ± SEM, and statistical significance was assessed by one‐way ANOVA followed by Tukey's post‐test (^*^ *p* < 0.05, ^****^ *p* < 0.0001).

At the end of these treatments, the tumors were subjected to histological evaluation of Ki67 proliferative marker, which is commonly used to assess the degree of tumor arrest in senescence‐induced therapy. As shown in **Figure** **6**A (first row), while the proliferating tumors showed high expression of Ki67 (brown staining), the expression decreased in the palbociclib‐treated tumors (Figure S12C, Supporting Information). For the palbocilib, palbociclib + **1**, and palbociclib + **1** + laser groups, there were no significant differences in the Ki67 expression, which suggested the occurrence of complete tumor arrest after the treatment with palbocilib, which is a characteristic feature of senescent tumors. Furthermore, we confirmed that the tumor arrest persisted and there was no enrichment of proliferating cells after the palbociclib administration was ceased. Besides, the activation of **1** in senescent tumors was confirmed by confocal microscopy. As shown in Figure 6A (second and third rows) and 6B (left), the fluorescence intensities of **1** for palbociclib + **1** and palbociclib + **1** + laser groups were more than 2‐fold higher than those for the vehicle groups, showing that **1** was activated by the overexpressed SA‐β‐gal in the senescent tumors. Finally, the in vivo PDT efficacy of **1** in killing senescent cells was also evaluated by counting the number of apoptotic cells in the tumor sections using the TUNEL assay. It was found that a noticeable signal could only be observed for the group of palbociclib + **1** + laser, which indicated the occurrence of extensive cell death through apoptosis [Figure 6A (fourth and fifth rows) and Figure 6B (right)]. The overall results demonstrate the selective activation of **1** in senescent cells, supporting the hypothesis that in the presence of overexpressed β‐gal in senescent cells, the *N*‐glycosidic bond of **1** is hydrolyzed, resulting in the release of the activated photosensitizer **2**. The controlled restoration of the fluorescence emission and single oxygen generation enables **1** to possess a precise and robust senolytic behavior.

## 3 Conclusion

In summary, a novel SA‐β‐gal‐activatable photosensitizer (compound **1**) consisting of a meso‐pyridinium BODIPY core conjugated with a galactose moiety through a self‐immolative linker is reported. The photoactivity of **1** is quenched, yet activation with β‐gal results in restoration of fluorescence emission and singlet oxygen generation. The ability of **1** to efficiently detect and kill senescent cells upon light irradiation is confirmed in vitro in chemotherapy‐induced senescent SK‐ Mel‐103 and HeLa cells. Moreover, the PDT efficacy of **1** is validated in vivo using a clinically relevant palbociclib‐induced senescent melanoma mouse model. Interestingly, **1** exhibits a remarkable therapeutic effect against the palbociclib‐induced senescent tumors, in which the fluorescence of BODIPY is restored and senolysis is triggered upon light irradiation. The results show that **1** is a promising agent for senescent cell elimination and one of the few examples of senolytic theranostic systems based on PDT. As mentioned above, compared to other therapies, PDT has several advantages and is a promising alternative for senotherapy due to its high spatiotemporal feature and reduced cytotoxicity with the added value that the photosensitizers can also be used as fluorescent probes for senescent cell detection. Given the increasing need of senolytic agents for selective elimination of senescent cells, our study is an attempt to address this need by developing a novel photosensitizer that can be specifically activated in senescent cells for PDT. This is one of the first reports validating a senolytic system based on PDT in a clinically relevant scenario. We believe that our study could inspire further design of a new generation of senolytic drugs whose therapeutic effect is triggered by light or other external stimuli.

## Conflict of Interest

The authors declare no conflict of interest.

## Supporting information

Supporting Information

## Data Availability

The data that support the findings of this study are available from the corresponding author upon reasonable request.
