# Supplementary material for: β‐Galactosidase‐Triggered Photodynamic Elimination of Senescent Cells with a Boron Dipyrromethene‐Based Photosensitizer
Source: Adv Sci (Weinh). 2024 Jun 17;11(31):2401012. doi: 10.1002/advs.202401012 (PMC11336962; doi:10.1002/advs.202401012)
Supplement: Supplementary file 1 — Supporting Information [file ADVS-11-2401012-s001.pdf]

ADVANCED SCIENCE Open Access

Supporting Information

for *Adv. Sci.*, DOI 10.1002/advs.202401012

β-Galactosidase-Triggered Photodynamic Elimination of Senescent Cells with a Boron Dipyrromethene-Based Photosensitizer

Jacky C. H. Chu, Blanca Escriche-Navarro, Junlong Xiong, Alba García-Fernández, Ramón Martínez-Máñez* and Dennis K. P. Ng**

Supporting Information

β-Galactosidase-Triggered Photodynamic Elimination of Senescent Cells with a Boron Dipyrromethene-Based Photosensitizer

Jacky C. H. Chu+, Blanca Escriche-Navarro+, Junlong Xiong+, Alba García-Fernández, Ramón Martínez-Máñez,* and Dennis K. P. Ng**

---

[*] Dr. J. C. H. Chu+, Dr. J. Xiong+, Prof. D. K. P. Ng

Department of Chemistry

The Chinese University of Hong Kong

Shatin, N.T., Hong Kong (China)

E-mail: dkpn@cuhk.edu.hk

B. Escriche-Navarro+, Dr. A. García-Fernández, Prof. R. Martínez-Máñez

Instituto Interuniversitario de Investigación de Reconocimiento, Molecular y Desarrollo Tecnológico

Universitat Politècnica de València, Universitat de València

Valencia 46022 (Spain)

E-mail: algarfe4@etsia.upv.es

rmaez@qim.upv.es

B. Escriche-Navarro+, Prof. R. Martínez-Máñez

Unidad Mixta de Investigación en Nanomedicina y Sensores

Universitat Politècnica e València, Instituto de Investigación Sanitaria La Fe (IIS La Fe)

Valencia 46026 (Spain)

B. Escriche-Navarro[+], Dr. A. García-Fernández, Prof. R. Martínez-Máñez

Unidad Mixta UPV-CIPF de Investigación en Mecanismos de Enfermedades y Nanomedicina

Universitat Politècnica de València, Centro de Investigación Príncipe Felipe

Valencia 46012 (Spain)

B. Escriche-Navarro[+], Dr. A. García-Fernández, Prof. R. Martínez-Máñez

CIBER de Bioingeniería, Biomateriales y Nanomedicina (CIBER-BBN), Instituto de Salud Carlos III

Madrid 28029 (Spain)

Dr. J. Xiong[+]

Department of Pharmacy

The Affiliated Luohu Hospital of Shenzhen University, Shenzhen University

Shenzhen 518001 (China)

[+] These authors contributed equally to this work.

Contents

Experimental Section

Figure S1 ^{1}H (top) and $^{13}C\{^{1}H\}$ (bottom) NMR spectra of **4** in DMSO-d_6.

Figure S2 ^{1}H (top) and $^{13}C\{^{1}H\}$ (bottom) NMR spectra of **1** in DMSO-d_6.

Figure S3 ESI mass spectrum of **4**.

Figure S4 ESI mass spectrum of **1**.

Figure S5 (A) Pseudo first-order kinetic plot of the reaction of **1** (2 μM) and β-gal (10 unit mL^{-1}) in PBS at pH 7.4 with Tween 80 (0.1% v/v). (B) Lineweaver-Burk plot for the kinetic study of the reaction of different concentrations of **1** and β-gal (10 unit mL^{-1}) in PBS at pH 7.4 with Tween 80 (0.1% v/v).

Figure S6 HPLC chromatograms of **1** in RPMI 1640 medium with FBS (10% v/v) at 37 ^{o}C at different time points.

Figure S7 Fluorescence spectra of **1** (2 μM) with or without the treatment with β-gal (10 unit mL^{-1}) for 2 h in RPMI 1640 medium with FBS (10% v/v) and Tween 80 (0.1% v/v) (λ_{ex} = 500 nm).

Figure S8 (A) HPLC chromatograms of **1**, the reaction mixtures after treating **1** (2 µM) with β-gal (10 unit mL^{-1}) in PBS with Tween 80 (0.1% v/v) for 1 and 2 h, and **2**. (b) ESI mass spectrum of the fraction with a retention time of 39.5 min.

Figure S9 (A) Bright field, fluorescence, and the merged images of proliferating and senescent HeLa cells after incubation with C_{12}FDG (25 µM) for 35 min, and comparison of the corresponding intracellular fluorescence intensities as determined by flow cytometry. Scale bar represents 100 μm. (B) Comparison of the intracellular fluorescence intensities in proliferating and senescent HeLa cells after incubation with **1** (2 μM) for 2 h with or without post-incubation in a fresh medium for 2 or 4 h as determined by flow cytometry. Data are reported

as the mean ± standard error of the mean (SEM) of three independent experiments, and statistical significance was assessed by t-test (****p <0.0001).

Figure S10 Confocal fluorescence images of senescent (A) SK-Mel-103 and (B) HeLa cells after costaining with **1** (in green) and MitoTracker or ER Tracker (in red). Areas of co-localization appear in yellow/orange in the Merge panel. Pearson's correlation coefficient (Rr) reported therein represents a correlation between the pixel intensity of **1** and that of the tracker in the enlarged image. The graphs on the right represent fluorescence intensity profiles along the white line drawn through the cells. Scale bar represents 20 μm for SK-Mel-103 cells and 50 μm for HeLa cells.

Figure S11 Intracellular ROS production as reflected by the quantified fluorescence intensity of the oxidized form of CellROX Deep Red Reagent in proliferating and senescent (A) SK-Mel-103 and (B) HeLa cells after being treated with **1** (0.5 μM for SK-Mel-103 cells and 2 μM for HeLa cells) for 2 h, followed by dark or light [SK-Mel-103 cells: λ > 475 nm, 14.3 mW cm^{-2}; HeLa cells: λ > 515 nm, 25.5 mW cm^{-2}] treatment for 5 min as determined by flow cytometry. Data are reported as the mean ± SEM of three independent experiments, and statistical significance was assessed by two-way ANOVA followed by Tukey's post-test (****p <0.0001).

Figure S12. (A) (Left) Change in tumor volume of SK-Mel-103 xenografts during the daily treatment with palbociclib (50 mg kg^{-1}) dissolved in 50 mM sodium lactate at pH 4.5 or simply the vehicle via oral gavage from day 4 to 11 (n = 4 mice per group). (Right) Photograph of representative tumor samples for each treatment after being stained with X-gal. (B) Change in body weight of the mice after different treatments over a period of 15 days. (C) Expression of Ki67 in the SK-

Mel-103 xenografts after different treatments. Data are reported as the mean ± SEM (n = 4), and statistical significance was assessed by one-way ANOVA followed by Dunnett's post-test (*p <0.05, **p <0.01).

Experimental Section

General

All the solvents and reagents were of reagent grade and used as received. All the reactions were carried out under an atmosphere of nitrogen and monitored by thin layer chromatography (TLC) performed on Merck pre-coated silica gel 60 F254 plates. Chromatographic purification was performed with column chromatography on silica gel (Macherey-Nagel, 230-400 mesh). Compounds **2**[R1] and **3**[R2] were prepared as described.

^{1}H and $^{13}C\{^{1}H\}$ NMR spectra were recorded on a Bruker AVANCE III 400 MHz spectrometer (^{1}H: 400 MHz, ^{13}C: 100.6 MHz) in DMSO-d_6. Spectra were referenced internally by using the residual solvent (^{1}H: δ = 2.50 ppm) or solvent (^{13}C: δ = 39.5 ppm) resonance relative to $SiMe_4$. Electrospray ionization (ESI) mass spectra were recorded on a Thermo Finnigan MAT 95 XL mass spectrometer. Electronic absorption and steady-state fluorescence spectra were taken on a Cary 5G UV-Vis-NIR spectrophotometer and a HORIBA FluoroMax-4 spectrofluorometer, respectively.

Liquid chromatography - mass spectrometry (LCMS) studies were performed on a XSelect CSH C18 column (5 μm, 4.6 mm × 250 mm) at a flow rate of 0.8 mL min^{-1} using a Waters system equipped with a Waters Quaternary Solvent Manager-R, a Waters 2998 photodiode array detector, and a Waters single quadrupole detector 2. The solvents used were of LCMS grade. The condition used for the analysis was set as follows: solvent A = 0.01% formic acid in acetonitrile and solvent B = 0.01% formic acid in deionized water; gradient: 100% A + 0% B in the first 5 min, changed to 0% A + 100% B in 30 min, maintained under this condition for 10 min, and then changed to 100% A + 0% B in 15 min.

Preparation of 4

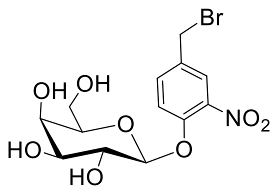

A mixture of **3** (2.52 g, 4.48 mmol) and K_2CO_3 (3.10 g, 22.4 mmol) in MeOH/CH_2Cl_2 (1:4 v/v, 40 mL) was stirred at room temperature overnight. The solvent was then evaporated under reduced pressure, and the residue was purified by chromatography on silica gel with MeOH/CH_2Cl_2 (1:8 v/v) as the eluent to afford **4** as a white solid (1.52 g, 86%). ^{1}H NMR (400 MHz, DMSO-d_6): δ 7.96-7.98 (m, 1 H, Ar-H), 7.70-7.73 (m, 1 H, Ar-H), 7.40-7.44 (m, 1 H, Ar-H), 5.18 (br s, 1 H, OH), 5.06 (d, *J* = 7.6 Hz, 1 H, gal-H), 4.91 (br s, 1 H, OH), 4.75 (s, 2 H, $PhCH_2$), 4.68 (br s, 1 H, OH), 4.61 (br s, 1 H, OH), 3.70 (br s, 1 H, gal-H), 3.63-3.66 (m, 1 H, gal-H), 3.49-3.57 (m, 3 H, gal-H), 3.39-3.42 (m, 1 H, gal-H). ^{13}C{^{1}H} NMR (100.6 MHz, DMSO-d_6): δ 149.9, 140.2, 135.2, 132.0, 125.8, 117.8, 101.4, 76.3, 73.8, 70.4, 68.4, 60.7, 33.0. HRMS (ESI): *m/z* calcd for $C_{13}H_{16}BrNNaO_8$ $[M+Na]^+$: 415.9951, found: 415.9946.

Preparation of 1

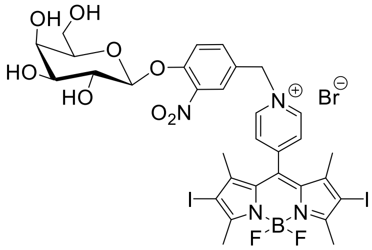

Compounds **4** (1.40 g, 3.54 mmol) and **2** (0.68 g, 1.18 mmol) were dissolved in CH_3CN (20 mL), and then the mixture was stirred under reflux for 12 h. After cooling, the mixture was filtered. The precipitate was washed with diethyl ether three times and then dried in vacuo to afford **1** as a deep red solid (0.26 g, 23%). ^{1}H NMR (400 MHz, DMSO-d_6): δ 9.44 (d, *J* = 6.8 Hz, 2 H, Ar-H), 8.48 (d, *J* = 6.8 Hz, 2 H, Ar-H), 8.10 (d, *J* = 2.0 Hz, 1 H, Ar-H), 7.87 (dd, *J* = 2.0, 8.8 Hz, 1 H, Ar-H), 7.53 (d, *J* = 8.8 Hz, 1 H, Ar-H), 6.01 (s, 2 H, $PhCH_2$), 5.22 (br s, 1 H,

OH), 5.12 (d, J = 7.6 Hz, 1 H, gal-H), 4.94 (br s, 1 H, OH), 4.67 (br s, 2 H, OH), 3.72 (d, J = 2.4 Hz, 1 H, gal-H), 3.69 (virtual t, J = 6.0 Hz, 1 H, gal-H), 3.54-3.61 (m, 2 H, gal-H), 3.46-3.50 (m, 1 H, gal-H), 3.41-3.44 (m, 1 H, gal-H), 2.58 (s, 6 H, CH_3), 1.39 (s, 6 H, CH_3). $^{13}C\{^1H\}$ NMR (100.6 MHz, DMSO-d_6): δ 158.4, 151.4, 150.7, 147.0, 144.9, 140.3, 135.1, 134.7, 129.6, 129.2, 127.8, 126.0, 118.0, 101.3, 88.8, 76.2, 73.8, 70.4, 68.4, 62.7, 60.7, 18.0, 16.5. HRMS (ESI): *m/z* calcd for $C_{31}H_{32}BF_2I_2N_4O_8^+$ $[M]^+$: 891.0370, found: 891.0359.

Determination of Fluorescence Quantum Yields

To minimize reabsorption of the radiation by the ground-state species, fluorescence spectra were recorded in very dilute solutions (2 μM). The fluorescence quantum yields (Φ_F) of the samples in PBS with Tween 80 (0.1% v/v) were determined by the equation:

$$\Phi_{F(sample)} = \left(\frac{F_{sample}}{F_{ref}}\right)\left(\frac{A_{ref}}{A_{sample}}\right)\left(\frac{n^2_{sample}}{n^2_{ref}}\right)\Phi_{F(ref)}$$

where F, A, and n are the measured fluorescence (area under the emission peak), the absorbance at the excitation wavelength (500 nm), and the refractive index of the solvent, respectively.[R3] Fluorescein in NaOH(aq) (0.1 M, pH 13) was used as the reference [$\Phi_{F(ref)} = 0.925$].[R4]

Determination of Singlet Oxygen Quantum Yields

9,10-Anthracenediyl-bis(methylene)dimalonic acid (ABDA) was used as a singlet oxygen scavenger. Compounds **1** and **2** (2 μM) were dissolved in PBS with Tween 80 (0.1% v/v), respectively, and the solutions were left at 37 °C for 2 h with or without β-gal (10 unit mL^{-1}) before ABDA (60 μM) was added. The resulting solutions were irradiated with light from a 100 W halogen lamp after passing through a water tank for cooling and a color filter with a cut-on wavelength at 515 nm (Newport). The absorbance of the ABDA's absorption at 400 nm was monitored along with the irradiation time. The values of singlet oxygen quantum yield (Φ_Δ) were calculated by the equation:[R5]

$$\Phi_{\Delta(\text{sample})} = \left(\frac{W^{sample}\, I_{\text{abs}}^{\text{ref}}}{W^{\text{ref}}\, I_{abs}^{sample}}\right) \Phi_{\Delta(\text{ref})}$$

where W and I_{abs} are the photobleaching rate of ABDA and the rate of light absorption, respectively, using rose bengal ($\Phi_\Delta = 0.75$) as the reference.[R6]

Study of Enzymatic Kinetics[R7]

The pseudo first-order rate constant for the reaction of **1** (2 μM) and β-gal (10 unit mL^{-1}) was determined by fitting the fluorescence intensities of the reaction mixture at different time points to the following pseudo first-order equation:

$$\ln\left(\frac{F_{max}-F_t}{F_{max}}\right) = -k't$$

where F_t, F_{max}, and k' are the measured fluorescence intensities at 567 nm at time point t, the maximum fluorescence intensity after the reaction was completed, and the pseudo first-order rate constant, respectively.

For the determination of Michaelis-Menten constant (K_m), the time-dependent fluorescence intensities of the reaction mixture of different concentrations of **1** and β-gal (10 unit mL^{-1}) were measured. The initial reaction velocities were then calculated. The relationship between the rate of enzymatic reaction and the concentration of **1** can be expressed by the Michaelis-Menten equation:

$$V_o = \left(\frac{V_{max}[S]}{K_m+[S]}\right)$$

where V_o, V_{max}, $[S]$, and K_m are the initial velocity, maximum velocity, substrate concentration, and Michaelis-Menten constant, respectively. K_m can be measured by using the Lineweaver-Burk plot with the following equation:

$$\frac{1}{V_o} = \frac{K_m}{V_{max}} \cdot \frac{1}{[S]} + \frac{1}{V_{max}}$$

where the slope is $\frac{K_m}{V_{max}}$ and y-intercept is $\frac{1}{V_{max}}$.

Study of the Stability of 1 in Serum

Compound **1** was dissolved in Roswell Park Memorial Institute (RPMI) 1640 medium (Invitrogen, cat. no. 23400-021) with fetal bovine serum (FBS) (ThermoFisher Scientific, cat. no. 10270-106) (10% v/v) at 37 °C. An aliquot of the mixture was drawn at different time points and precipitated with an equal volume of cold methanol. The mixture was centrifuged, and the supernatant was collected for HPLC analysis.

Cell Culture and Senescence Induction

Human melanoma SK-Mel-103 cell line was obtained from ATCC and maintained in Dulbecco's Modified Eagle Medium (DMEM) supplemented with FBS (10% v/v). For senescence induction, SK-Mel-103 cells were cultured in the medium containing 5 μM palbociclib (Selleckchem, cat. no. S1116) for 7 days. Human cervical adenocarcinoma HeLa cells (ATCC, no. CCL-2) were maintained in RPMI 1640 medium (Invitrogen, cat. no. 23400-021) supplemented with FBS (10% v/v) and penicillin-streptomycin (100 unit mL^{-1} and 100 μg mL^{-1}, respectively). For senescence induction, HeLa cells were cultured in the medium containing 50 nM doxorubicin (DOX) for 3 days. All the cells were grown at 37 ºC in a humidified 5% CO_2 atmosphere.

Senescence-Associated β-Galactosidase (SA-β-gal) Activity Assays

SA-β-gal detection was carried out using the β-galactosidase senescence staining kit (Cell Signaling, cat. no. 9860S) according to the manufacturer's instructions. Briefly, cells were washed with PBS and fixed at room temperature for 12 min with a fixative solution (2% formaldehyde and 0.2% glutaraldehyde in PBS). Following fixation, the cells were washed with PBS three times, stained with a X-gal staining solution in *N*,*N*-dimethylformamide (pH

6.0), and incubated overnight at 37 °C without CO_2. A Leica DM6000 inverted microscope was then used to detect the blue staining of the cells. To detect SA-β-gal in tumors, the whole tumors were fixed with 4% paraformaldehyde overnight. Subsequently, the tumors were washed with PBS and incubated with a X-gal staining solution for 4 h at 37 °C.

The increased level of β-gal in the senescent HeLa cells was also verified with C_{12}FDG. Approximately 1 × 10^4 HeLa cells per well in RPMI 1640 medium (2 mL) were seeded on a 6-well plate and incubated overnight at 37 °C in a humidified 5% CO_2 atmosphere. After removal of the medium, the cells were rinsed with PBS (1 mL) and incubated in RPMI 1640 medium containing DOX (50 nM) for 3 days. The cells were rinsed with PBS (1 mL) twice and then incubated in a DOX-free RPMI 1640 medium for a further 24 h. After being rinsed with PBS, the senescent cells were prepared for the subsequent studies. For the proliferating HeLa cells, approximately 1 × 10^5 HeLa cells in RPMI 1640 medium (2 mL) were seeded on a confocal dish and incubated overnight at 37 °C in a humidified 5% CO_2 atmosphere. The cells were incubated with C_{12}FDG (25 μM) in a serum-free RPMI 1640 medium for 35 min at 37 °C. After being rinsed with PBS twice, the cells were examined using a Leica TCS SP8 high-speed confocal microscope equipped with a 488 nm laser. The dye was excited at 488 nm and the fluorescence was monitored at 500−600 nm. The images were digitized and analyzed using Leica Application Suite X software. To quantify the β-gal activity by flow cytometry, the medium was removed, and the cells were rinsed with PBS twice and harvested by 0.25% trypsin-ethylenediaminetetraacetic acid (Invitrogen, 0.2 mL) for 5 min. The activity of trypsin was quenched with a serum-containing medium (0.5 mL), and the mixture was centrifuged at 1500 rpm for 3 min at room temperature. The pellet was then washed with PBS (1.0 mL) and then subjected to centrifugation. The cells were suspended in PBS (1.0 mL) and the intracellular fluorescence intensities were measured using a BD FACSVerse flow cytometer (Becton Dickinson) with 10^4 cells counted in each sample. The compound was excited by an

argon laser at 488 nm, and the emitted fluorescence was monitored at 500−600 nm. The data collected were analyzed using the BD FACSuite. All experiments were performed in triplicate.

Cellular Internalization and Activation Studies

One day before performing the assay, proliferating SK-Mel-103 cells (2.5 × 10^5 cells), palbociclib-treated (5 μM for 7 days) senescent SK-Mel-103 cells (1.5 × 10^5 cells), proliferating HeLa cells (1 × 10^5 cells), and DOX-treated (50 nM for 3 days) senescent HeLa cells were seeded. A stock solution of **1** (1 mM) was prepared by dissolving **1** (20 nmol) in DMSO (20 μL). The cells were incubated with **1** (2 μM) for 2 h at 37 ^{o}C. The solutions were then removed, and the cells were rinsed with PBS twice before being examined with a Leica TCS SP8 high-speed confocal microscope equipped with a 488 nm laser. The dye was excited at 488 nm and the fluorescence was monitored at 500−600 nm. The images were digitized and analyzed using Leica Application Suite X software.

Subcellular Localization Studies

Senescent SK-Mel-103 and HeLa cells were incubated with **1** (2 μM) at 37 ^{o}C for 2 h. After being rinsed with PBS twice, the cells were stained with different organelle-specific markers, namely LysoTracker Deep Red (Thermo Fisher Scientific Inc., cat. no. L12492) (0.1 μM for 30 min), MitoTracker Red CMXRos (Thermo Fisher Scientific Inc., cat. no. M7512) (0.2 μM for 15 min), ER-Tracker Red (Thermo Fisher Scientific Inc., cat. no. E34250) (1 μM for 15 min), and ER-Tracker Blue-White DPX (Thermo Fisher Scientific Inc., cat. no. E12353) (0.5 μM for 30 min), respectively, at 37 °C. Subsequently, the cells were examined with a Leica TCS SP8 high-speed confocal microscope equipped with a 405 nm laser, a 488 nm laser, a 552 nm laser, and a 638 nm laser using a 63X objective lens. The parameters of the laser scanning confocal microscope (CLSM) were set as follows: LysoTracker Deep Red was excited at 638

nm, and the fluorescence was monitored at 650−680 nm; MitoTracker Red CMXRos and ER-Tracker Red were excited at 552 nm, and their fluorescence was monitored at 590−620 nm; ER-Tracker Blue-White DPX was excited at 405 nm and its emission was collected at 410−480 nm. Compound **1** was excited at 488 nm and its fluorescence was monitored at 500−600 nm. The images were digitized and analyzed using Leica Application Suite X software.

Photocytotoxicity Assay

Approximately 1×10^3 HeLa cells per well in RPMI 1640 medium were inoculated in 96-well plates and incubated overnight at 37 °C in a humidified 5% CO_2 atmosphere. After removal of the medium, the cells were rinsed with PBS and incubated in RPMI 1640 medium containing DOX (50 nM, 200 μL) for 3 days. The cells were rinsed with PBS twice and then incubated in a DOX-free RPMI 1640 medium (100 μL) for a further 24 h. After being rinsed with PBS, the senescent cells were prepared for the subsequent studies. For the proliferating HeLa cells, approximately 1×10^4 HeLa cells per well in RPMI 1640 medium were inoculated in 96-well plates and incubated overnight at 37 °C in a humidified 5% CO_2 atmosphere. A stock solution of **1** (1 mM) was prepared as described above, which was then diluted with a serum-free medium to the respective concentrations. The cells, after being rinsed with PBS twice, were incubated with 100 μL of the solutions of **1** at 37 °C for 2 h. The cells were then rinsed again with PBS and refed with 100 μL of the culture medium, followed by the dark or light treatment for 20 min. For the latter, the light source consisted of a 300 W halogen lamp, a water tank for cooling, and a color glass filter (Newport) cut-on at $\lambda = 515$ nm. The fluence rate ($\lambda > 515$ nm) was 25.5 mW cm^{-2}. Irradiation for 20 min led to a total fluence of 30.6 J cm^{-2}. Cell viability was determined by the CellTiter-Glo luminescent cell viability assay.[R8] After illumination, the cells were incubated at 37 °C under 5% CO_2 overnight. A solution of CellTiter-Glo reagent (Promega) (100 μL) was added to each well and the solutions in all wells were mixed on an

orbital shaker to induce cell lysis. The plate was incubated at room temperature for 10 min to stabilize the luminescent signal. The luminescent signal of each well on the plate was taken by a microplate reader (Tecan Spark 10M) at ambient temperature. The average luminescence of the blank wells, which did not contain the cells, was subtracted from the readings of the other wells. The cell viability was then determined by the equation:

$$\% \text{ viability} = \frac{\Sigma\left(\frac{A_{\mathrm{i}}}{A_{\mathrm{control}}}\right)\times 100}{n},$$

where A_i is the luminescence of the ith datum (i = 1, 2, …, n), $A_{control}$ is the average luminescence of the control wells in which the compound was absent, and n (= 4) is the number of data points.

For SK-MEL-103 cells, the cytotoxic effect of **1** was determined using a WST-1 cell proliferation and viability assay (Roche, cat. no. 11644807001).[R9] Proliferating and senescent SK-MEL-103 cells were seeded in 96-well plates at a density of 7.5×10^3 and 5×10^3 cells per well, respectively, and incubated for 24 h at 37 ^{o}C under 5% CO_2. The cells were then treated with different concentrations of **1**, ranging from 0.1 to 5 μM, prepared in DMEM (containing 1% DMSO). After incubation for 2 h, the cells were rinsed with PBS and illuminated with a 36 W LED device with a colored glass filter (Newport) with cut-off at λ = 475 nm (14.3 mW cm^{-2}). A total fluence of 25.7 J cm^{-2} was obtained by irradiation for 30 min. After further incubation for 24 h, 10 μL of WST-1 was added to each well and the plates were incubated for 1 h at 37 ^{o}C. The absorbance at 450 nm of the samples was measured with a Wallac 1420 Victor2 microplate reader (Perkin Elmer). The toxicity of the compound in the dark was evaluated under the same conditions as above but omitting the illumination step. The IC_{50} value (i.e., the concentration of compound that is required to reduce 50% of cell viability compared to the untreated control) was estimated from a sigmoidal dose-response curve fit of the photocytotoxicity data from at least three independent analyses, using GraphPad Prism 9.

Study of Intracellular ROS Generation

Proliferating and senescent SK-Mel-103 and HeLa cells were incubated with **1** for 2 h (0.5 μM for SK-Mel-103 cells and 2 μM for HeLa cells). After being rinsed with PBS twice, the cells were stained with 5 μM of CellROX Deep Red Reagent (Thermo Fisher Scientific Inc., cat. no. C10422) for 30 min at 37 °C. After that, the cells were subjected to dark or light treatment using the aforementioned light sources for 5 min. The total fluence was 4.3 J cm^{-2} for SK-Mel-103 cells and 7.7 J cm^{-2} for HeLa cells. The cells were then examined with a Leica TCS SP8 high-speed confocal microscope using a 63X objective lens. The fluorescent product localized in the cytoplasm, generated by the oxidation of the reagent by ROS, was excited at 638 nm and its fluorescence was monitored at 650−680 nm. The images were digitized and analyzed using Leica Application Suite X software.

Apoptosis Assay with Annexin V-APC/DAPI

Proliferating SK-Mel-103 cells (1×10^5 cells per well) and senescent SK-Mel-103 cells (5.5×10^4 cells per well) were cultured in 12-well plates and allowed to settle for 24 h. The medium was then changed to a fresh medium containing **1** at a concentration of 0.5 μM. After 2 h, the cells were washed with PBS and irradiated for 30 min ($\lambda > 475$ nm, 14.3 mW cm^{-2}, 25.7 J cm^{-2}). After incubation for a further 24 h, the treated cells, both floating and adherent, were collected, centrifuged (300 x g, 5 min, 4 °C), and suspended in 0.2 mL of Annexin V binding buffer (1X) containing 5 μL of Annexin V conjugated with allophycocyanin (APC). After incubation for 15 min at 4 °C in dark, 1 μL of 4',6-diamidino-2-phenylindole (DAPI) (1 mg mL^{-1}) was added to each sample. The cells were then determined whether they were viable (Annexin V-negative, DAPI-negative), early apoptotic (Annexin V-positive, DAPI-negative), late apoptotic/necrotic (Annexin V-positive, PI-positive), or necrotic (Annexin V-negative,

DAPI-positive) by flow cytometry (CytoFlexS instrument, Beckman Coulter). Data analysis was performed with CytoExpert software.

In Vivo PDT Experiments

All mice were treated in strict accordance with the Ethical Committee for Research and Animal Welfare Generalitat Valenciana, Conselleria dÀgricultura, Medi ambient Canvi climàtic i Desenvolupament Rural (2022 VSC PEA 193). To induce senescence in tumor, 5 x 10^5 SK-Mel-103 cells were injected subcutaneously into 8–10-week-old athymic nude mice (Hsd: Athymic Nude-Foxn1nu, Envigo) in two flanks. When the tumor volume reached ca. 50 mm^3 on day 4, daily treatment with palbociclib (50 mg kg^{-1}) dissolved in 50 mM sodium lactate at pH 4.5 or simply the vehicle via oral gavage was initiated. These treatments were maintained for 7 days, and on day 11, the animals were euthanized in a CO_2 atmosphere and the tumors were harvested for subsequent evaluation.

To evaluate the senolytic activity of **1** by PDT in SK-Mel-103 xenograft, 5 x 10^5 SK-Mel-103 cells were injected subcutaneously into 8–10-week-old athymic nude mice (Hsd: Athymic Nude-Foxn1nu, Envigo) in two flanks. The animals were randomly divided into six different groups receiving different treatments: (1) vehicle; (2) vehicle + **1**; (3) vehicle + **1** + laser; (4) palbociclib; (5) palbociclib + **1**, and (6) palbociclib + **1** + laser. Each group contained five mice (n = 10 tumors per group). Tumors were measured with calipers every 2 days, and tumor volume (mm^3) was calculated with the formula length x $width^2/2$. When the tumor volume reached ca. 50 mm^3 on day 4, daily treatment with palbociclib (50 mg kg^{-1}) dissolved in 50 mM sodium lactate at pH 4.5 or simply the vehicle via oral gavage was initiated to induce senescence from day 4 to day 10 both included. On day 11, compound **1** (40 nmol) was administered intratumorally, and the tumors were irradiated after 2 h with a 525 nm laser for 5 min (500 mW cm^{-2}, 150 J cm^{-2}). The effect of the different treatment groups was monitored by

measuring the tumor size and body weight of the mice. At the end of the treatments, the animals were euthanized in a CO_2 atmosphere and the tumors were harvested for subsequent evaluation.

Histological Analysis

Tumors that were fixed with 4% paraformaldehyde at 4 °C overnight were washed with PBS three times and then cut into halves. The tumor halves were incubated with 30% sucrose at 4 °C overnight, embedded in cryomolds with OCT, and frozen completely at -20 °C. To measure the fluorescence of **1** in tumor sections, tumors were cut into 10 μm cryosections, mounted on glass slides, and stained with DAPI in dark. Images were obtained with a Leica TCS SP8 high-speed confocal microscope using a 40X objective lens. The mean fluorescence intensity of **1** was quantified using ImageJ software. To evaluate apoptosis, TUNEL staining was performed following the manufacturer's instructions of the In Situ Cell Death Detection Kit (Merck, cat. no. 12156792910). Subsequently, the sections were stained with DAPI and covered with a glass coverslip using a mounting medium. Confocal microscopic images were obtained with a Leica TCS SP8 high-speed confocal microscope using a 40X objective lens, and the positive signal was quantified using CellProfiler software.

The other halves of the tumors were embedded in paraffin for immunohistological Ki67 staining. The 5 mm paraffin sections were deparaffinized and rehydrated. Antigen retrieval was then performed using 10 mM sodium citrate buffer and 0.05% Tween 20 at pH 6.0 for 30 min at 97 ºC. To inactivate the endogenous peroxidase, the slides were incubated with Dako REAL Peroxidase-Blocking Solution (S2023) for 10 min at room temperature. The tumor sections were then incubated in a blocking solution (5% horse serum, 0.3% Triton X-100 in 1 × PBS) for 1 h and incubated with Ki67 antibody (Cell Signaling, cat. no. 12202; 1:400) at 4 °C overnight. The next day, the slides were incubated with a biotinylated rabbit HRP secondary antibody (Abcam, cat. no. ab205718; 1:200) for 1 h at room temperature in a humidified

chamber. Ki67 immunostaining was revealed with 3,3-diaminobenzidine tetrahydrochloride (DAB), and nuclei were counterstained with hematoxylin. Sections were scanned on a Leica Aperio Versa 200 at 20x magnification.

Statistical Analysis

Data are presented as the mean ± standard error of the mean (SEM) processed with GraphPad Prism 9 software. Statistical analysis was performed with Student's t-test, one-way ANOVA or two-way ANOVA (analysis of variance), followed by a Tukey's or Dunnett's post-test, as indicated in the figure legends. Statistical significance is indicated as *p <0.05, **p <0.01, ***p <0.001, and ****p <0.0001. Only significant statistical differences are indicated in the graphs.

[R1] B. Yuan, H. Wang, J.-F. Xu, X. Zhang, *ACS Appl. Mater. Interfaces* **2020**, *12*, 26982−26990.

[R2] J. Xiong, J. C. H. Chu, W.-P. Fong, C. T. T. Wong, D. K. P. Ng, *J. Am. Chem. Soc.* **2022**, *144*, 10647–10658.

[R3] D. F. Eaton, *Pure Appl. Chem.* **1988**, *60*, 1107−1114.

[R4] J. C. T. Carlson, L. G. Meimetis, S. A. Hilderbrand, R. Weissleder, *Angew. Chem. Int. Ed.* **2013**, *52*, 6917–6920; *Angew. Chem.* **2013**, *125*, 7055–7058.

[R5] S. E. Maree, T. Nyokong, *J. Porphyrins Phthalocyanines* **2001**, *5*, 782−792.

[R6] G. Linden, L. Zhang, F. Pieck, U. Linne, D. Kosenkov, R. Tonner, O. Vázquez, *Angew. Chem. Int. Ed.* **2019**, *58*, 12868–12873; *Angew. Chem.* **2019**, *131*, 13000–13005.

[R7] Z. Li, M. Ren, L. Wang, L. Dai, W. Lin, *Sens. Actuators B: Chem*, **2020**, *307*, 127643.

[R8] B. Lozano-Torres, I. Galiana, M. Rovira, E. Garrido, S. Chaib, A. Bernardos, D.

Muñoz-Espín, M. Serrano, R. Martínez-Máñez, F. Sancenón, *J. Am. Chem. Soc.* **2017**, *139*, 8808−8811.

[R9] S. Kamiloglu, G. Sari, T. Ozdal, E. Capanoglu, *Food Front.* **2020**, *1*, 332–349.

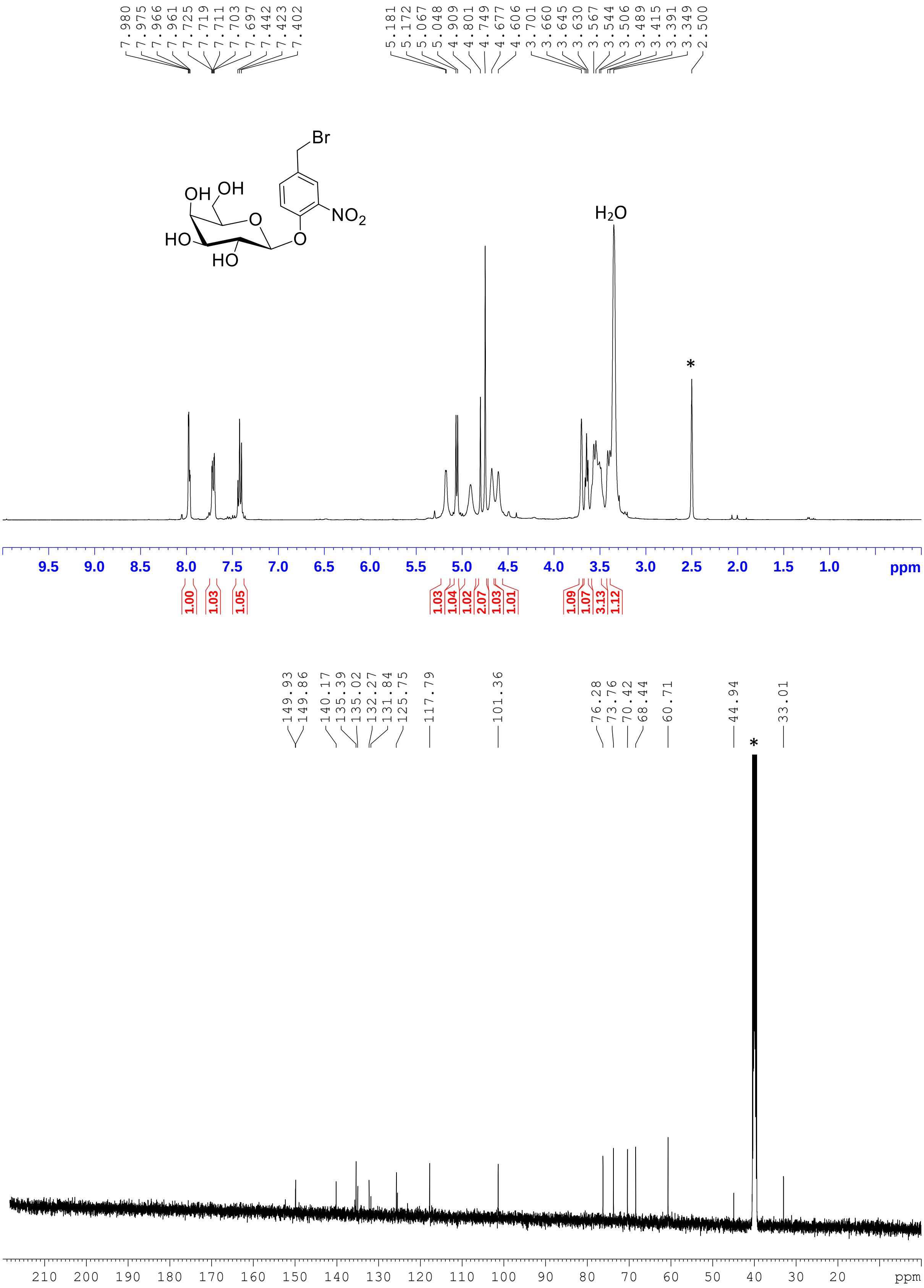

Figure S1. ^{1}H (top) and $^{13}C\{^{1}H\}$ (bottom) NMR spectra of **4** in DMSO-d_6.

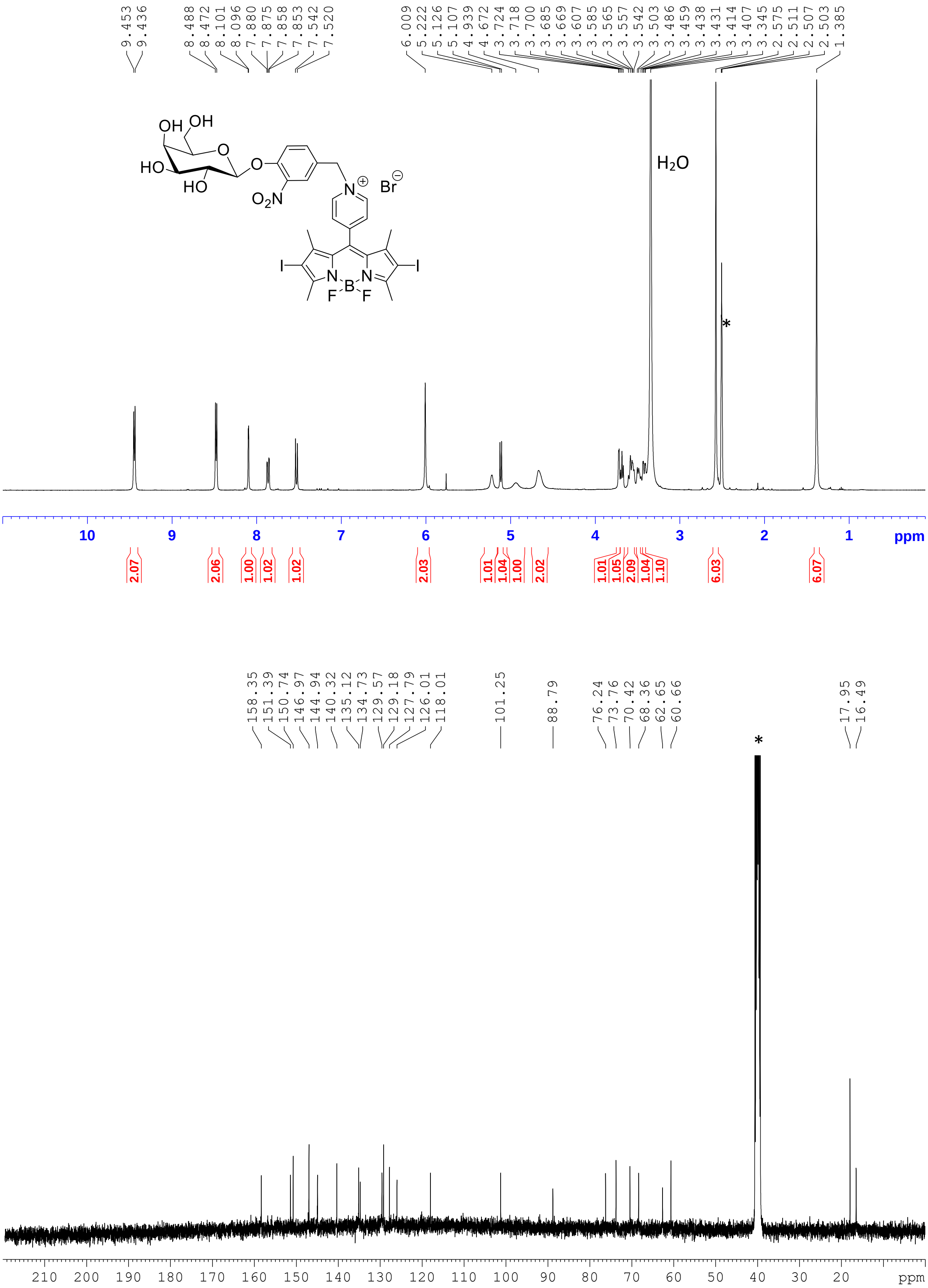

Figure S2. ^{1}H (top) and $^{13}C\{^{1}H\}$ (bottom) NMR spectra of **1** in DMSO-d6.

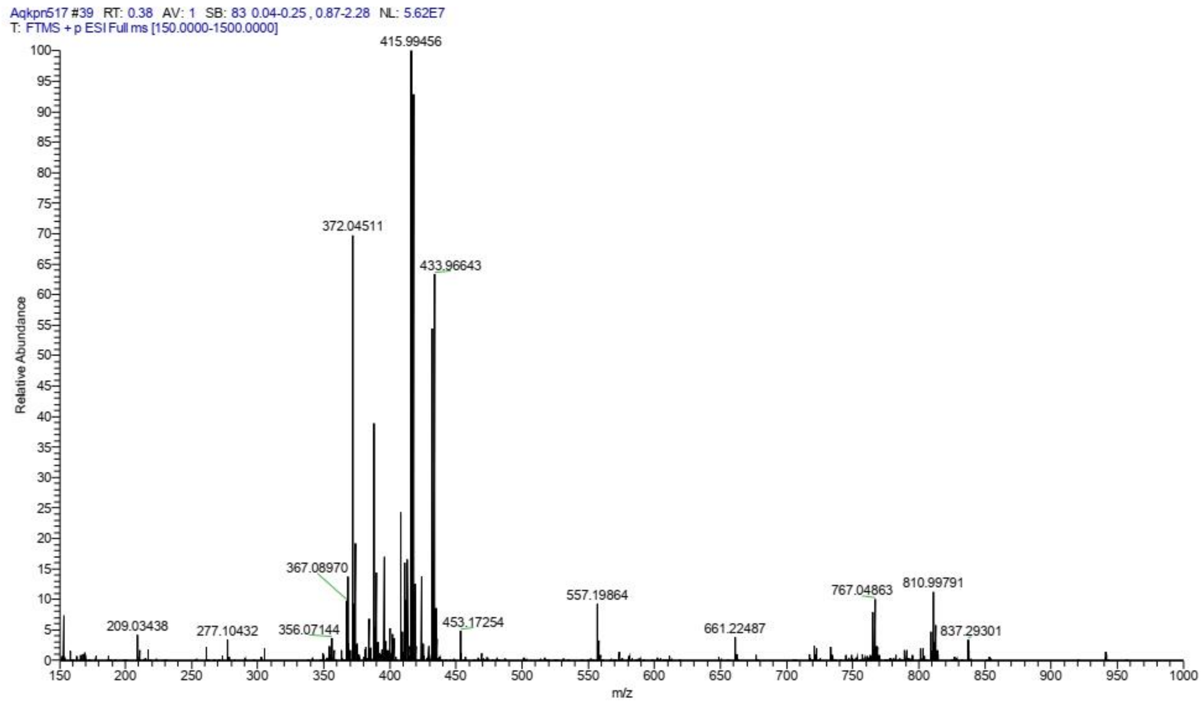

Figure S3. ESI mass spectrum of **4**.

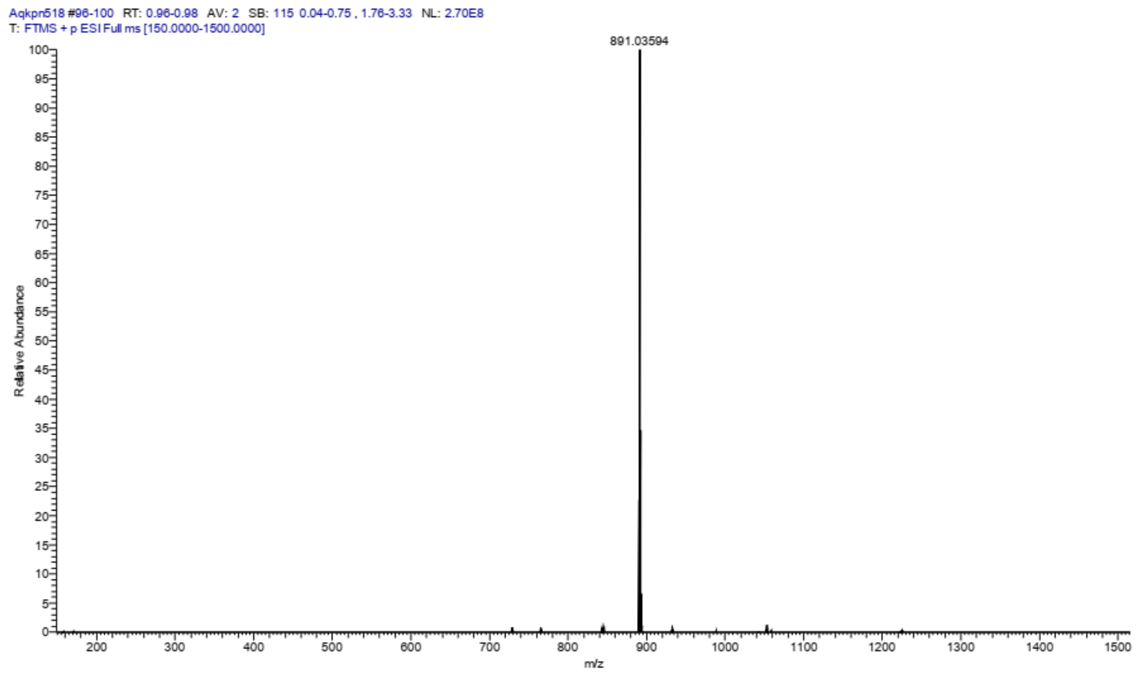

Figure S4. ESI mass spectrum of **1**.

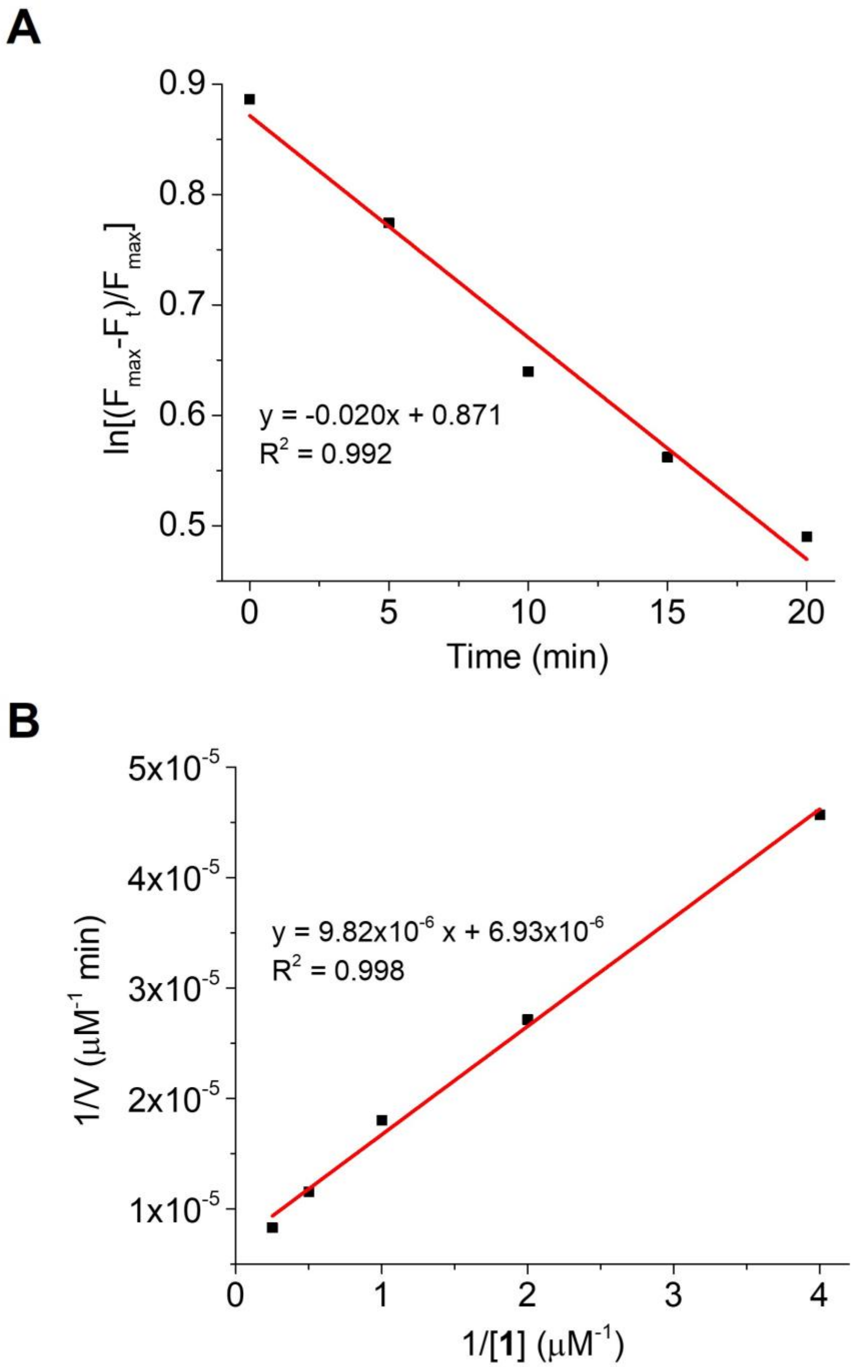

Figure S5. (A) Pseudo first-order kinetic plot of the reaction of **1** (2 μM) and β-gal (10 unit mL^{-1}) in PBS at pH 7.4 with Tween 80 (0.1% v/v). (B) Lineweaver-Burk plot for the kinetic study of the reaction of different concentrations of **1** and β-gal (10 unit mL^{-1}) in PBS at pH 7.4 with Tween 80 (0.1% v/v).

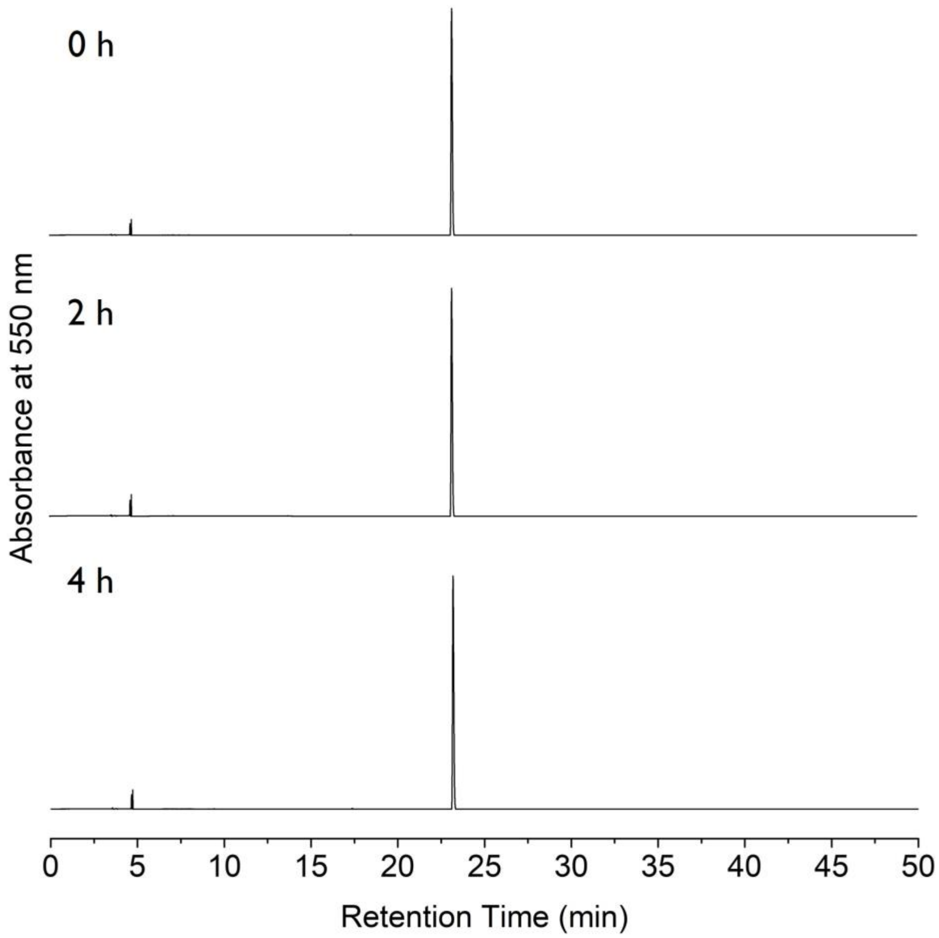

Figure S6. HPLC chromatograms of **1** in RPMI 1640 medium with FBS (10% v/v) at 37 ^{o}C at different time points.

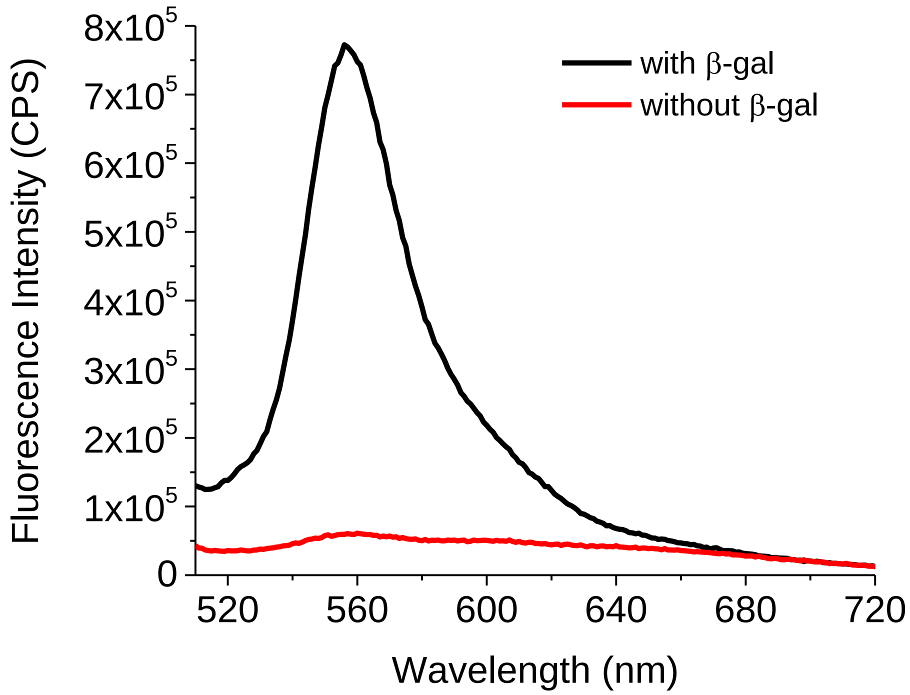

Figure S7. Fluorescence spectra of **1** (2 μM) with or without the treatment with β-gal (10 unit mL^{-1}) for 2 h in RPMI 1640 medium with FBS (10% v/v) and Tween 80 (0.1% v/v) (λ_{ex} = 500 nm).

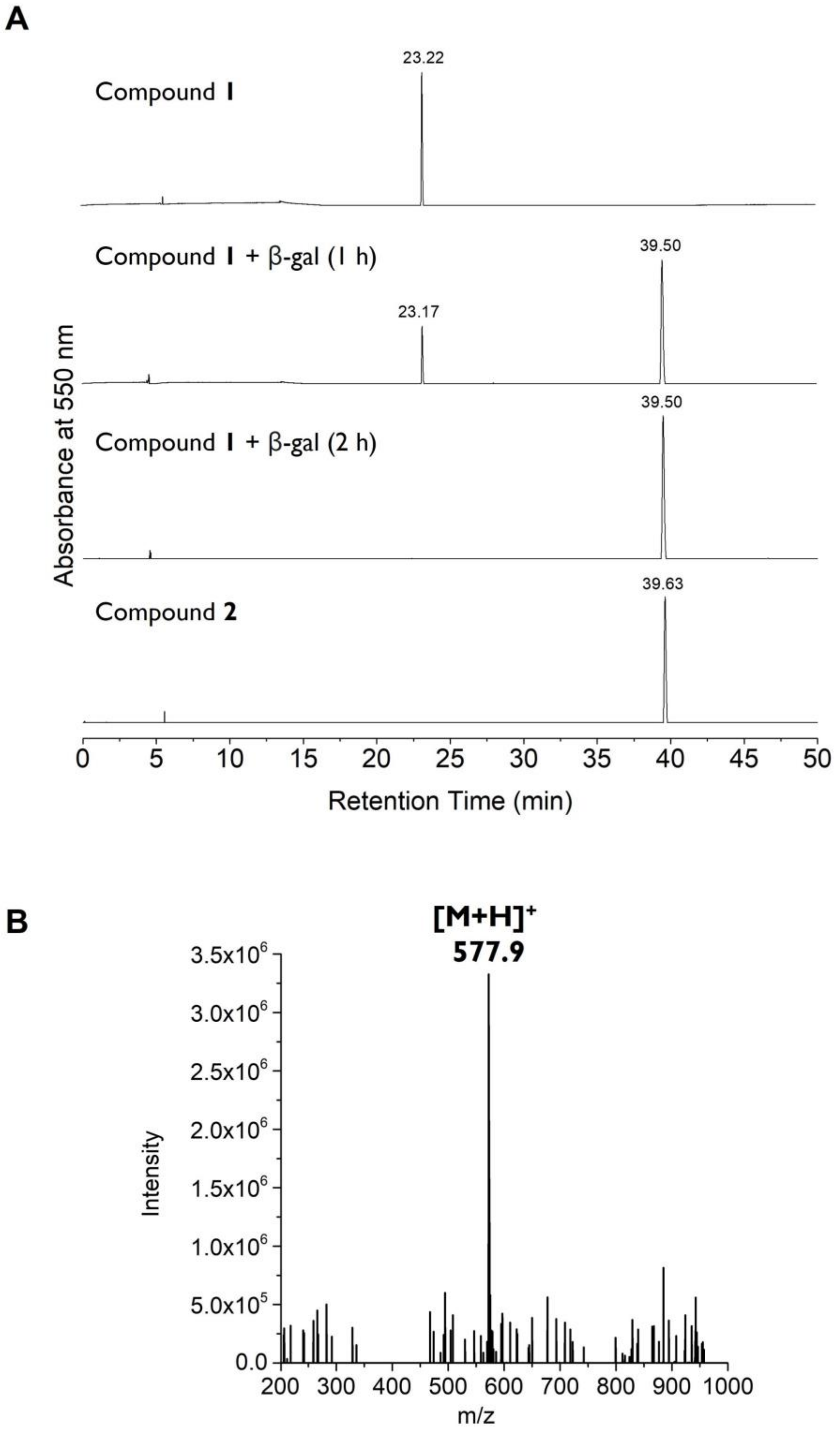

Figure S8. (A) HPLC chromatograms of **1**, the reaction mixtures after treating **1** (2 μM) with β-gal (10 unit mL^{-1}) in PBS with Tween 80 (0.1% v/v) for 1 and 2 h, and **2**. (b) ESI mass spectrum of the fraction with a retention time of 39.5 min.

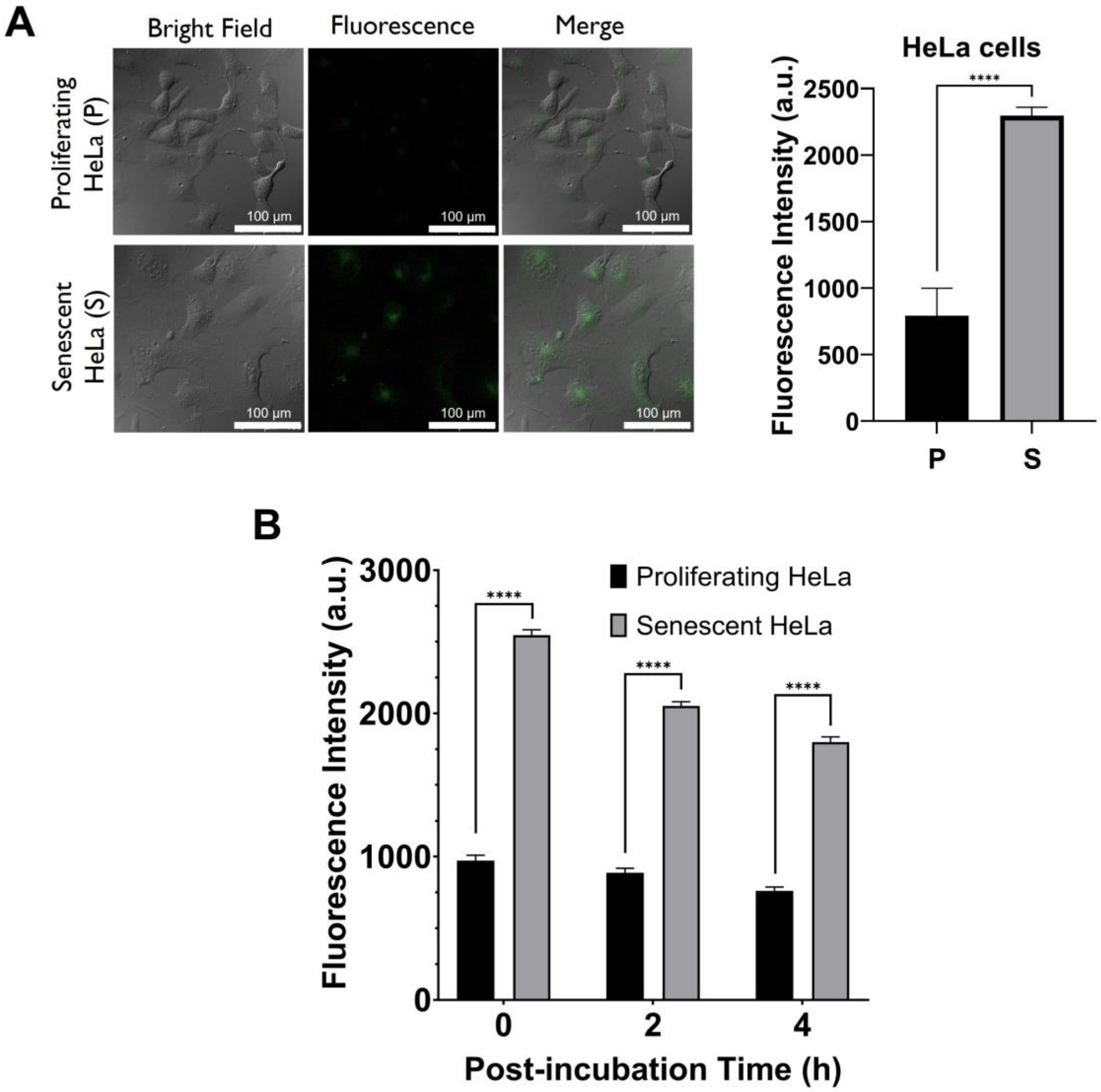

Figure S9. (A) Bright field, fluorescence, and the merged images of proliferating and senescent HeLa cells after incubation with C_{12}FDG (25 μM) for 35 min, and comparison of the corresponding intracellular fluorescence intensities as determined by flow cytometry. Scale bar represents 100 μm. (B) Comparison of the intracellular fluorescence intensities in proliferating and senescent HeLa cells after incubation with **1** (2 μM) for 2 h with or without post-incubation in a fresh medium for 2 or 4 h as determined by flow cytometry. Data are reported as the mean ± standard error of the mean (SEM) of three independent experiments, and statistical significance was assessed by t-test (****p <0.0001).

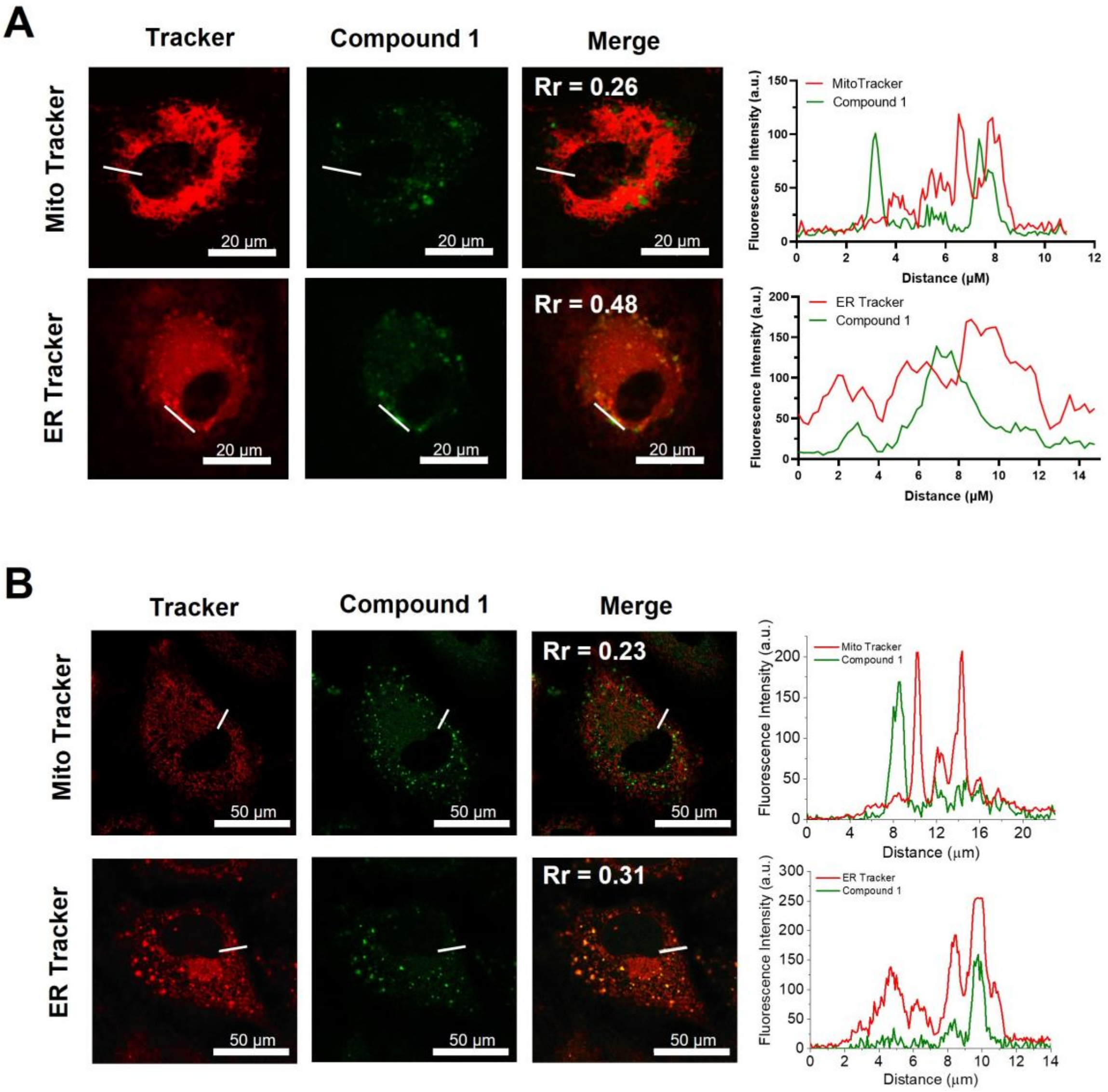

Figure S10. Confocal fluorescence images of senescent (A) SK-Mel-103 and (B) HeLa cells after costaining with **1** (in green) and MitoTracker or ER Tracker (in red). Areas of co-localization appear in yellow/orange in the Merge panel. Pearson's correlation coefficient (Rr) reported therein represents a correlation between the pixel intensity of **1** and that of the tracker in the enlarged image. The graphs on the right represent fluorescence intensity profiles along the white line drawn through the cells. Scale bar represents 20 µm for SK-Mel-103 cells and 50 µm for HeLa cells.

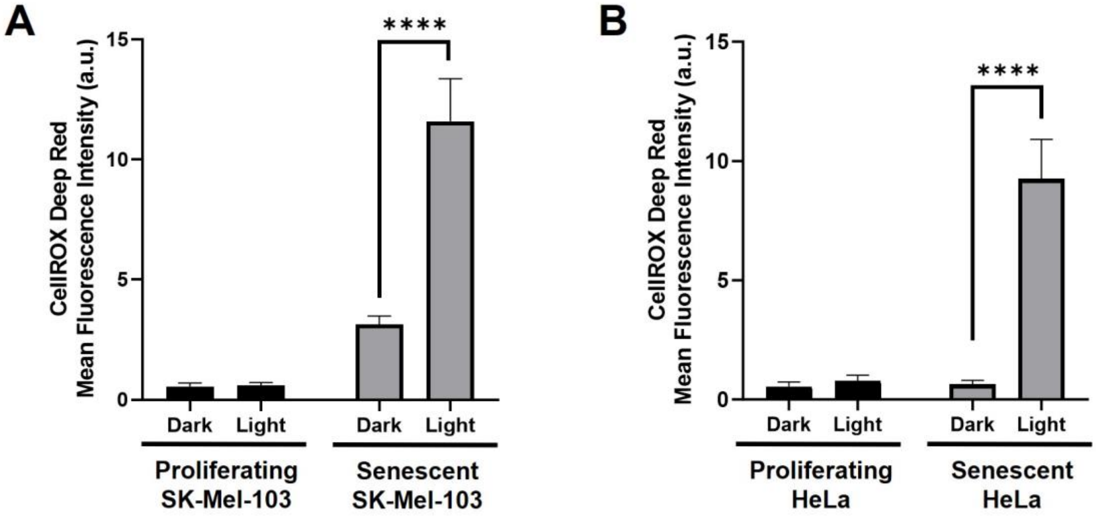

Figure S11. Intracellular ROS production as reflected by the quantified fluorescence intensity of the oxidized form of CellROX Deep Red Reagent in proliferating and senescent (A) SK-Mel-103 and (B) HeLa cells after being treated with **1** (0.5 μM for SK-Mel-103 cells and 2 μM for HeLa cells) for 2 h, followed by dark or light [SK-Mel-103 cells: $\lambda > 475$ nm, 14.3 mW cm^{-2}; HeLa cells: $\lambda > 515$ nm, 25.5 mW cm^{-2}] treatment for 5 min as determined by flow cytometry. Data are reported as the mean ± SEM of three independent experiments, and statistical significance was assessed by two-way ANOVA followed by Tukey's post-test (****$p < 0.0001$).

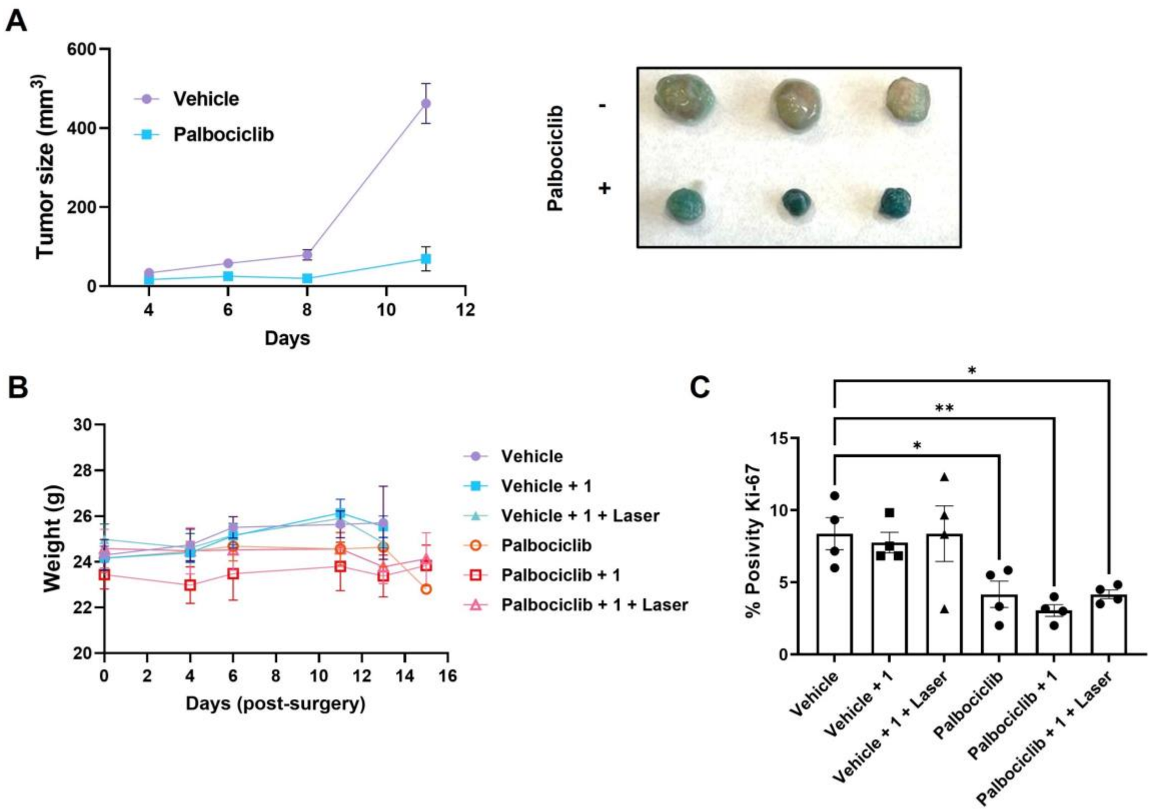

Figure S12. (A) (Left) Change in tumor volume of SK-Mel-103 xenografts during the daily treatment with palbociclib (50 mg kg^{-1}) dissolved in 50 mM sodium lactate at pH 4.5 or simply the vehicle via oral gavage from day 4 to 11 (n = 4 mice per group). (Right) Photograph of representative tumor samples for each treatment after being stained with X-gal. (B) Change in body weight of the mice after different treatments over a period of 15 days. (C) Expression of Ki67 in the SK-Mel-103 xenografts after different treatments. Data are reported as the mean ± SEM (n = 4), and statistical significance was assessed by one-way ANOVA followed by Dunnett's post-test (*p <0.05, **p <0.01).
